# Aerobic Oxidations in Asymmetric Synthesis: Catalytic Strategies and Recent Developments

**DOI:** 10.3389/fchem.2021.614944

**Published:** 2021-03-30

**Authors:** Dzmitry Kananovich, Gábor Zoltán Elek, Margus Lopp, Victor Borovkov

**Affiliations:** Department of Chemistry and Biotechnology, School of Science, Tallinn University of Technology, Tallinn, Estonia

**Keywords:** oxygen, aerobic oxidation, asymmetric synthesis, enantioselective catalysis, chirality, organocatalysis, transition metals

## Abstract

Despite the remarkable advances in the area of asymmetric catalytic oxidations over the past decades, the development of sustainable and environmentally benign enantioselective oxidation techniques, especially with the efficiency level similar to natural enzymes, still represents a challenge. The growing demand for enantiopure compounds and high interest to industry-relevant green technological advances continue to encourage the research pursuits in this field. Among various oxidants, molecular oxygen is ubiquitous, being available at low cost, environmentally benign and easy-to-handle material. This review highlights recent achievements in catalytic enantioselective oxidations utilizing molecular oxygen as the sole oxidant, with focus on the mechanisms of dioxygen activation and chirogenesis in these transformations.

## Introduction

In modern organic synthesis, asymmetric oxidations are of fundamental importance. The early works of Sharpless, (Katsuki and Sharpless, 1980; Jacobsen et al., 1988; Katsuki and Martin, 2004), Jacobsen (Zhang et al., 1990; Jacobsen et al., 1988) and Shi (Tu et al., 1996) on the stereoselective epoxidation and dihydroxylation of alkenes have established prominent benchmarks, with a strong impact to the whole field of enantioselective catalysis. Owing to the valuable contributions of numerous esteemed research groups, contemporary methods of asymmetric oxidation constitute a powerful and multifunctional tool for the preparation of versatile chiral molecules. However, widespread utilization of hazardous oxidants with poor atom economy (e.g. hypochlorites, iodine(III), and iodine(V) reagents, quinones, peroxy compounds) constitutes a serious shortcoming in the view of current green chemistry paradigm (Anastas and Warner, 1998).

As a consequence, the asymmetric catalytic oxidations with molecular oxygen as a terminal oxidant can be considered as the next Frontier in organic synthesis, (Mukaiyama and Yamada, 1995; Jiao and Stahl, 2019; Sterckx et al., 2019), being especially attractive for large-scale industrial applications (Caron et al., 2006; Hone et al., 2017). Dioxygen itself is an abundant compound presented in air (21% by volume) and vital for the metabolism of aerobic organisms, thus representing an inexpensive and eco-friendly oxidizing agent. It also offers excellent atom economy: as a rule, aerobic oxidation usually produces low molecular weight by-products (e.g. H_2_O) or even proceeds with 100% atom efficiency (e.g. formation of peroxides). Hence, atmospheric oxygen nicely fits the green chemistry requirements, being a nearly “ideal” oxidant.

Besides the mentioned green chemistry benefits, recent accomplishments in asymmetric synthesis with atmospheric oxygen often represent prominent examples in the catalytic reaction engineering. Indeed, to ensure reliable stereodiscrimination of a prochiral substrate and enhanced reactivity of oxygen, the design of enantioselective aerobic oxidation process should commonly comprise two main components: an effective oxygen activation pathway and a proper method of chirality induction (termed “chirogenesis”) (Borovkov et al., 2001).

**FIGURE 1:**
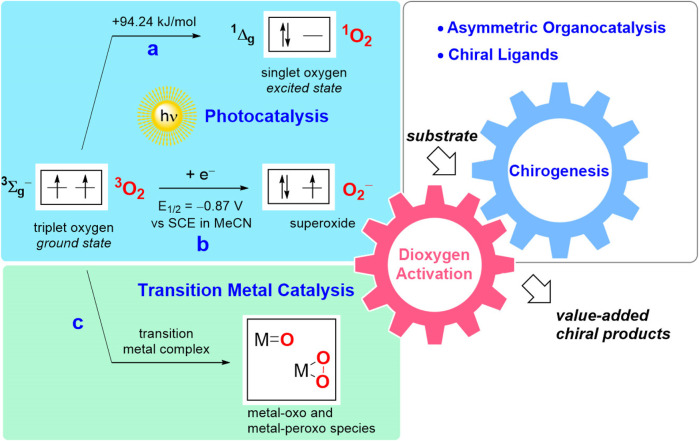
Schematic representation of O_2_ activation pathways and chirogenesis in enantioselective catalytic aerobic oxidations: **(A)** generation of singlet oxygen; **(B)** photoredox catalysis yielding a superoxide anion; **(C)** transition metal catalysis.

In its ground state, dioxygen molecule represents a stable biradical (triplet oxygen, ^3^O_2_). Although the oxidation of organic compounds is thermodynamically favorable, the reaction of triplet oxygen with usually singlet-state organic molecules is spin forbidden, hence imposing a kinetic limitation on this reaction pathway (Miller et al., 1990). As a consequence, the organic substrates can only be oxidized with triplet oxygen via a radical autoxidation process (Doering and Haines, 1954; Ingold, 1961; Porter, 2013; Poon and Pratt, 2018). Alternatively, transformation of ^3^O_2_ into another type of the oxidative species must be implemented to overcome the corresponding kinetic constraint. As one option, activation of triplet oxygen can be achieved with the aid of photocatalysis, resulting in generation of highly reactive singlet oxygen ^1^O_2_ (Figure 1A) (Zamadar and Greer, 2009; Ghogare and Greer, 2009). Another activation pathway is based on single electron transfer from a photoredox catalyst (Romero and Nicewicz, 2016) (or another monoelectronic reductant) yielding superoxide anion radical O_2_^−^ (Figure 1B). Furthermore, various transition metal complexes can shuttle oxygen atoms even at ambient conditions via generation of the corresponding oxo- and peroxo species, or transfer electrons in the oxidation reactions (Figure 1C) (Miller et al., 1990; Parmeggiani and Cardona, 2012; Allen et al., 2013). The chirogenic process in transition metal-catalyzed transformations is conventionally induced by a judicious choice of chiral ligand, while photocatalytic approaches are commonly merged with asymmetric organocatalysis.

It is remarkable that the mechanisms outlined above are also relevant for the operation of iron- (Meunier et al., 2004), copper- (Festa and Thiele, 2011), manganese- (Dismukes, 1996), and metal-free flavin-containing enzymes, which provide an inspiration for designing the analogs of the enzymatic reactions (de Gonzalo and Fraaije, 2013; Petsi and Zografos, 2018). Many of these transformations involve synergetic or dual catalytic cycles, featuring masterpieces in the catalytic reaction engineering.

The main aim of the current review is to outline the key catalytic strategies and latest innovative concepts in design of the asymmetric reactions with molecular oxygen as a terminal oxidant. Therefore, the most prominent examples have been judiciously selected among the research works published in the last 20 years. However, for a more detailed overview of the field, especially earlier contributions, a number of comprehensive reviews (Uchida and Katsuki, 2013; Bryliakov, 2015; Bryliakov, 2017; Ottenbacher et al., 2018), and personal accounts (Mukaiyama and Yamada, 1995; Irie and Katsuki, 2004) can be recommended for further reading.

The first part of the review describes transformations mediated by transition metals. Besides asymmetric oxidations mediated by chiral metal complexes, dual catalytic processes are also mentioned, in which the aerobic oxidation step is used solely to generate a prochiral intermediate for the subsequent asymmetric reaction. The second part focuses on metal-free aerobic oxidations, commonly operating via dual photo-organocatalytic cycles. It worth mentioning that enantioselective oxidations mediated by enzymes, (Endoma et al., 2002; Boyd and Bugg, 2006; Gandomkar et al., 2019), heterogeneous catalysts, (Miyamura et al., 2013), and nanoparticles (Miyamura et al., 2015; Hadian-Dehkordi and Hosseini-Monfared, 2016) also represent an important cluster of valuable synthetic methodologies, however these approaches are out of the scope of this review.

## Transition Metal-Catalyzed Transformations

**FIGURE 2:**
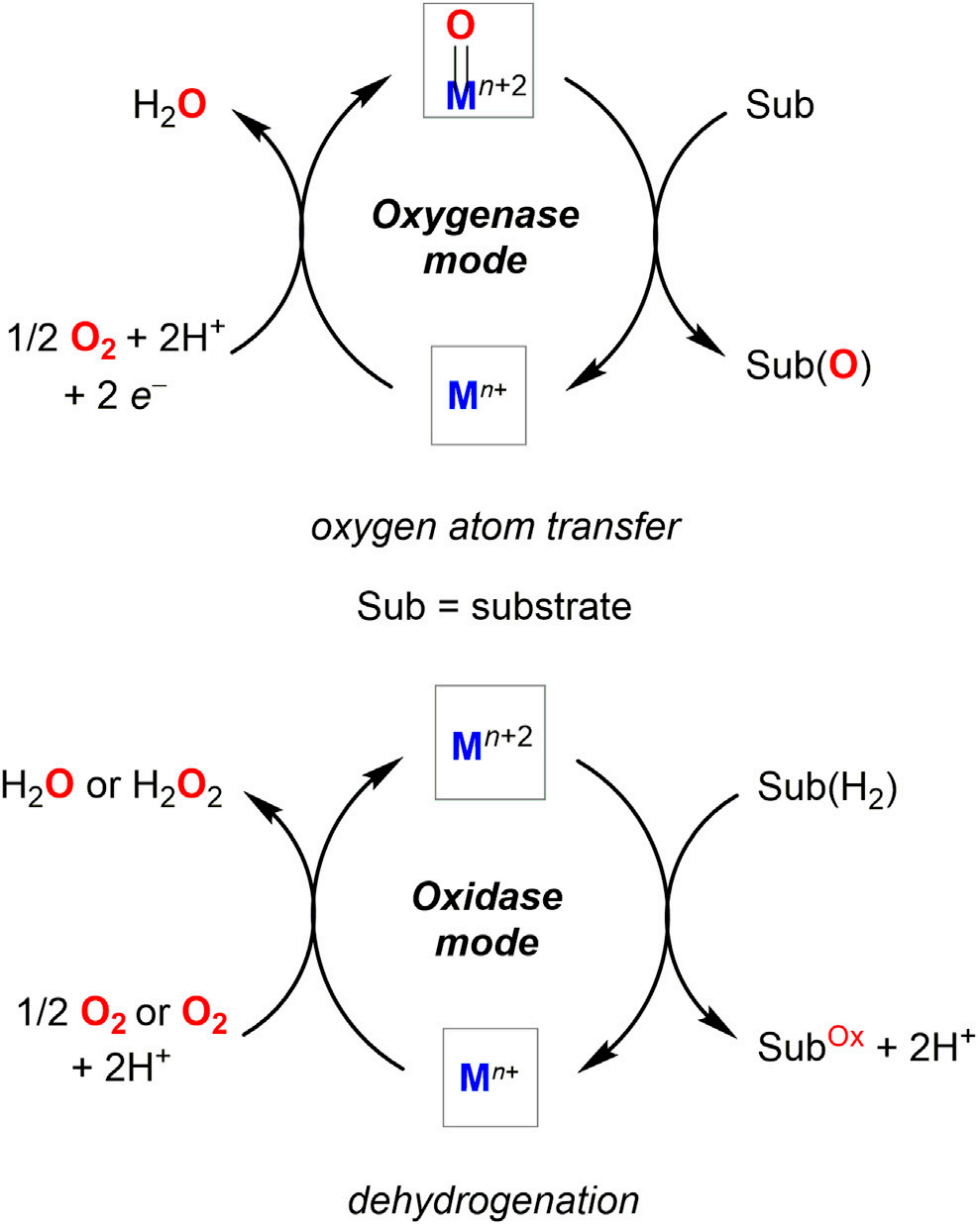
Oxygenase and oxidase pathways of transition metal-mediated aerobic oxidations.

Transition metal-catalyzed transformations constitute the mainstream approach in enantioselective aerobic oxidations. In general, the corresponding catalytic cycles mimic enzymatic activity of metal-containing oxidases and oxygenases (Figure 2), (Bugg, 2003; McCann and Stahl, 2015) with asymmetry typically induced by a chiral environment. Besides being an oxidant, transition metal can also act as a Lewis acid to enable substrate coordination and to facilitate stereodiscrimination of the enantiomeric pairs in oxidative kinetic resolution process.

Among the recent advances, synergetic transition metal-photocatalysis (Ding et al., 2017) can be mentioned as a more intricate reaction design, in which photochemically-generated singlet oxygen is exploited.

**FIGURE 3:**
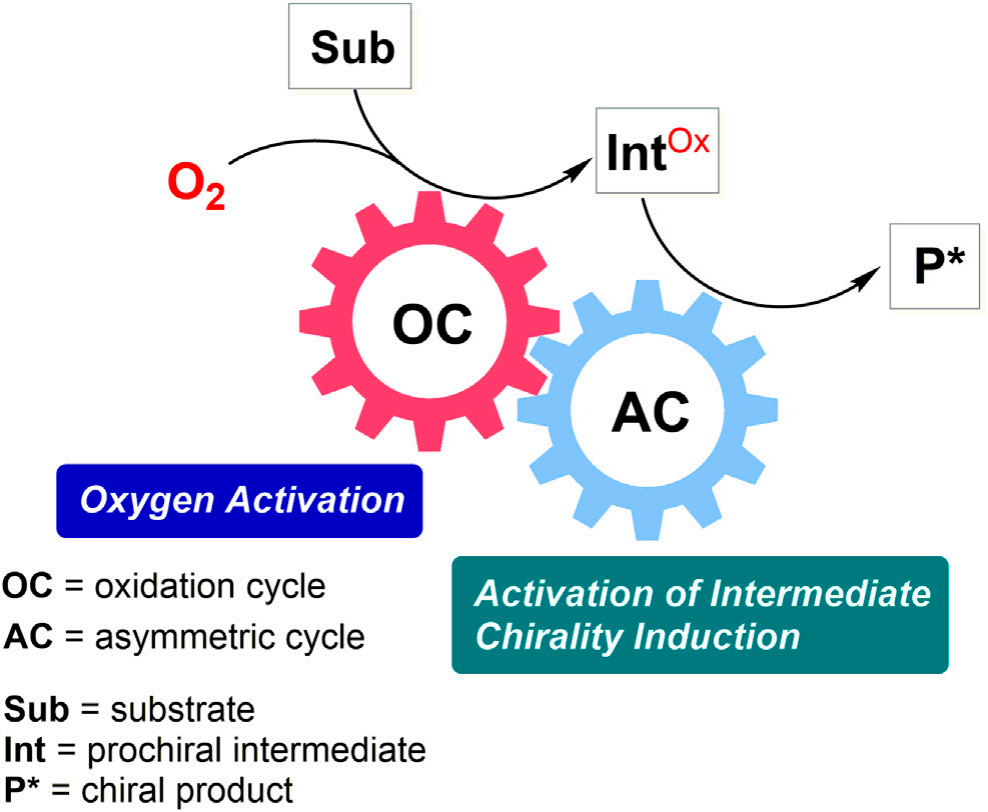
Dual catalytic enantioselective aerobic oxidation cascade.

The transformations presented in this section are subdivided according to the catalytic strategy and origin of chirogenesis. While the majority of enantioselective metal-catalyzed aerobic oxidations relies on the use of a single chiral transition metal catalyst, several cascade or dual catalytic transformations are also known, in which aerobic oxidation cycle is used solely to generate a reactive prochiral intermediate (Figure 3). In the latter approach, chirality is typically induced in an additional catalytic cycle, as presented in the second subsection.

### Asymmetric Aerobic Oxidations Mediated by Chiral Metal Complexes

Application of the transition metal complexes as chiral oxygen carriers in asymmetric aerobic oxidations was pioneered by the Mukaiyama’s group in 1990s, (Mukaiyama and Yamada, 1995) which designed a number of optically active Mn^III^ complexes to perform the enantioselective epoxidation of olefins (Yamada et al., 1992; Mukaiyama et al., 1993; Nagata et al., 1994; Yamada et al., 1994) and oxidation of sulfides to chiral sulfoxides (Imagawa et al., 1995; Nagata et al., 1995). These keynote contributions were followed by the discovery of chiral nickel (Kureshy et al., 1998) and ruthenium complexes, (Kureshy et al., 1997; Lai et al., 1998), which are capable to mediate the enantioselective aerobic epoxidation of olefins, and works of the Bolm’s group on the copper-catalyzed Baeyer-Villiger oxidation of cyclic ketones (Bolm et al., 1994; Bolm and Schlingloff, 1995; Bolm et al., 1997). It should be noted that these prominent early contributions have been extensively discussed in several reviews (Mukaiyama and Yamada, 1995; Bryliakov, 2017; Ottenbacher et al., 2018). The use of chiral metal complexes still represent a prevalent approach among the known aerobic oxidation methods.

**SCHEME 1:**
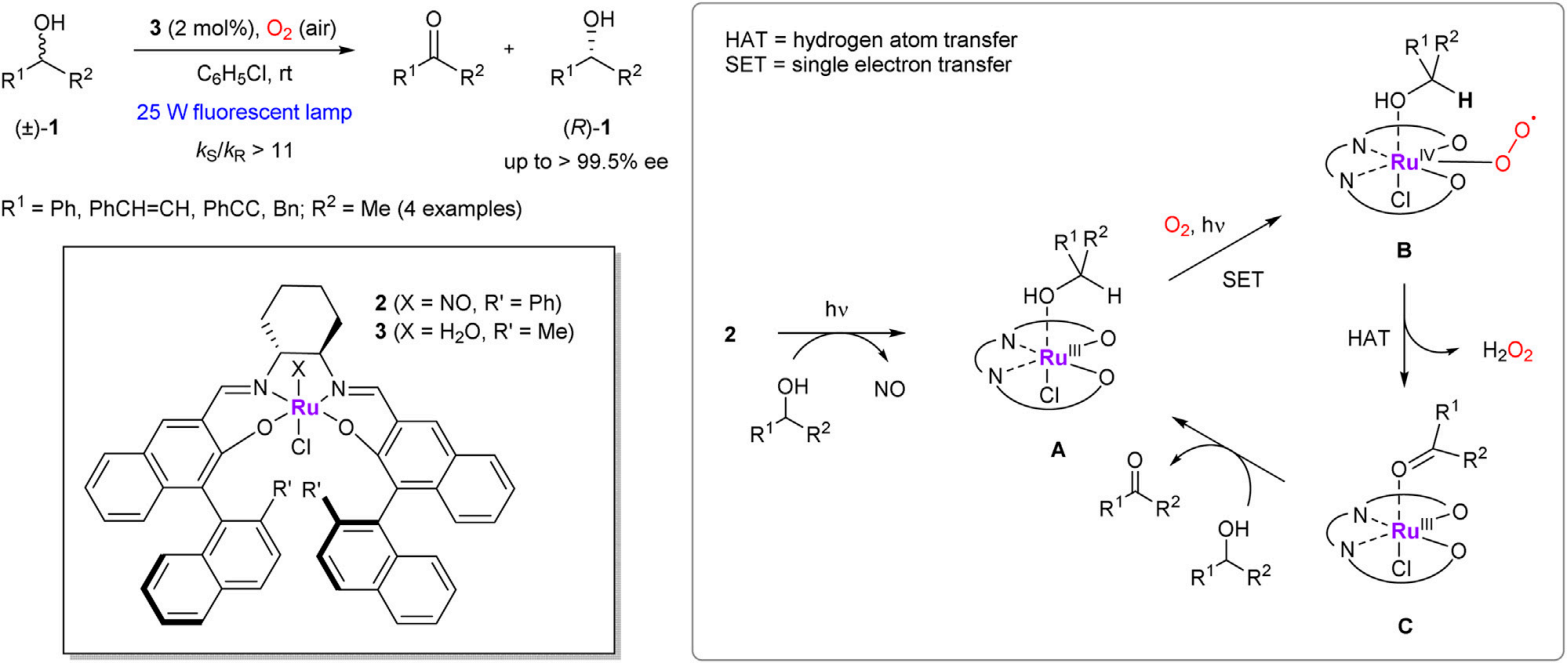
Aerobic oxidative kinetic resolution of secondary alcohols with Ru-salen complexes **2**, **3** and a simplified representation of its mechanism (Masutani et al., 2000; Shimizu et al., 2005).

In 2000, Katsuki et al. reported a highly efficient protocol for chemo- and enantioselective aerobic oxidation of secondary alcohols **1** under visible-light irradiation, mediated by optically active (nitroso)-salen ruthenium complex **2** (Scheme 1) (Masutani et al., 2000). The reaction mechanism was suggested on the basis of kinetics and kinetic isotope effect studies (Shimizu et al., 2005). It was suggested that irradiation triggers the dissociation of complex **2** and release of NO ligand, thus providing a free coordination site for the alcohol substrate. The produced Ru^III^ complex **A** captures molecular oxygen to afford Ru^IV^ peroxo complex **B**, where the peroxo ligand accepts α-carbynol hydrogen of the alcohol ligand via a hydrogen atom transfer (HAT) process. The resulting ketone-anchoring Ru^III^ species **C** finally recovers the complex **A** via replacement of the ketone ligand with the next molecule of alcohol. In a more recent study the same group demonstrated that a broad range of alcoholic substrates can be employed in aerobic oxidative kinetic resolution even without irradiation, just with the aid of (aqua)-salen ruthenium complex **3** (Mizoguchi et al., 2014). Applications of chiral (NO)Ru-salen complexes in enantioselective aerobic oxidations showed a great potential for further development and have been expanded far beyond the kinetic resolution of alcohols in subsequent works of the same group (Irie and Katsuki, 2004). Thus, similar ruthenium-based catalysts have been successfully used in the enantioselective oxidative couplings of 2-naphthols, (Irie et al., 2000), radical cyclization of 2,2′-dihydroxystilbene, (Masutani et al., 2002), and asymmetric desymmetrization of *meso*-diols (Shimizu et al., 2002; Shimizu and Katsuki, 2003).

**SCHEME 2:**
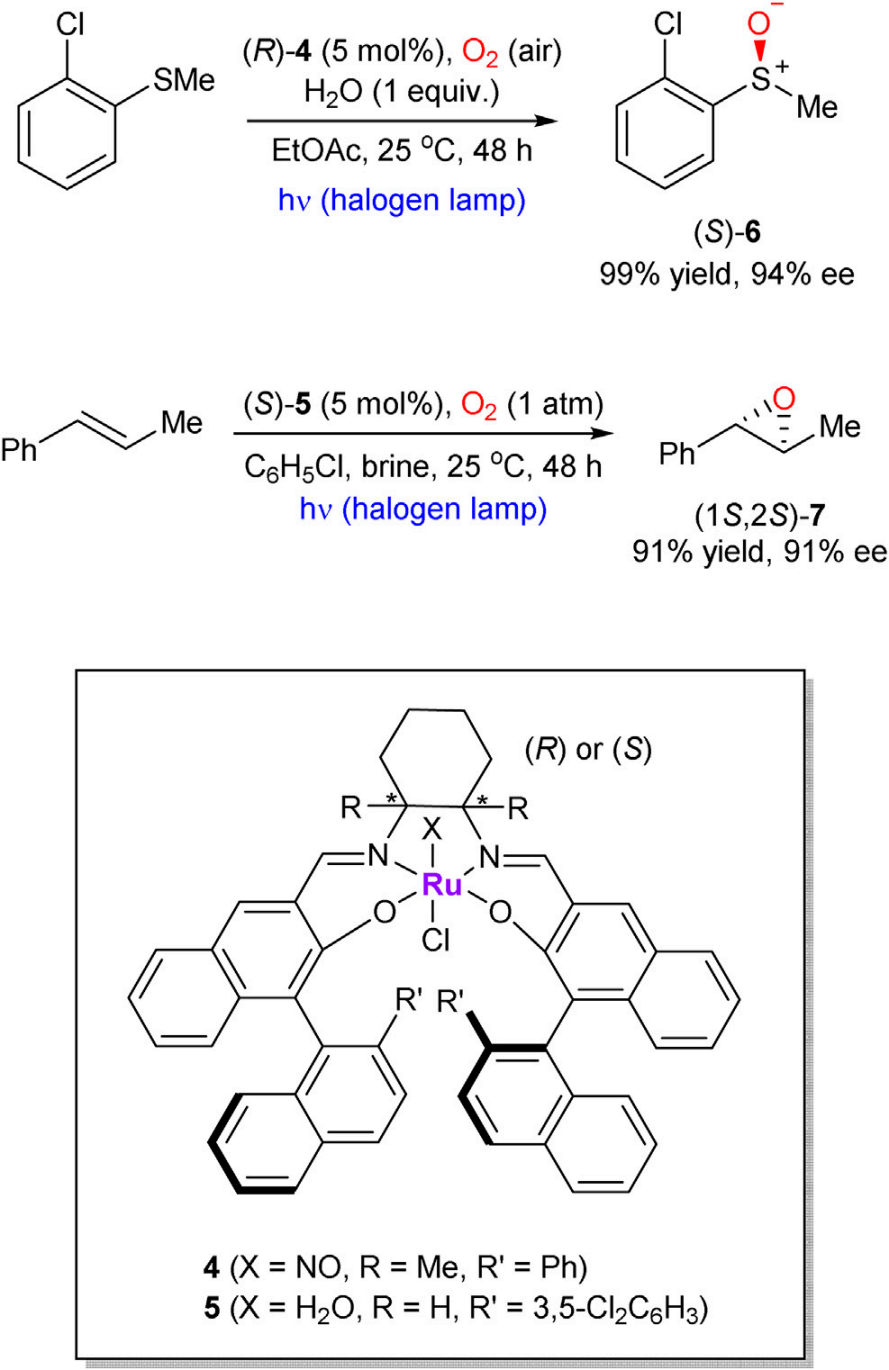
Photo-promoted Ru-catalyzed enantioselective sulfide oxidation and alkene epoxidation with molecular oxygen (Tanaka et al., 2010; Koya et al., 2012).

Katsuki’s chiral Ru-salen complexes are also capable to mediate the “oxygenase type” transformations, such as epoxidation of olefins and oxidation of sulfides (representative examples **6** and **7** are shown in Scheme 2) (Tanaka et al., 2010; Koya et al., 2012). In comparison with early works on the enantioselective Ru-catalyzed aerobic epoxidations of olefins, (Kureshy et al., 1997; Lai et al., 1998), Katsuki’s protocol did not require an aldehyde co-reductant or high oxygen pressure and operates at ambient conditions. Interestingly, water was found to be an essential additive, which serves as a proton transfer mediator.

**SCHEME 3:**
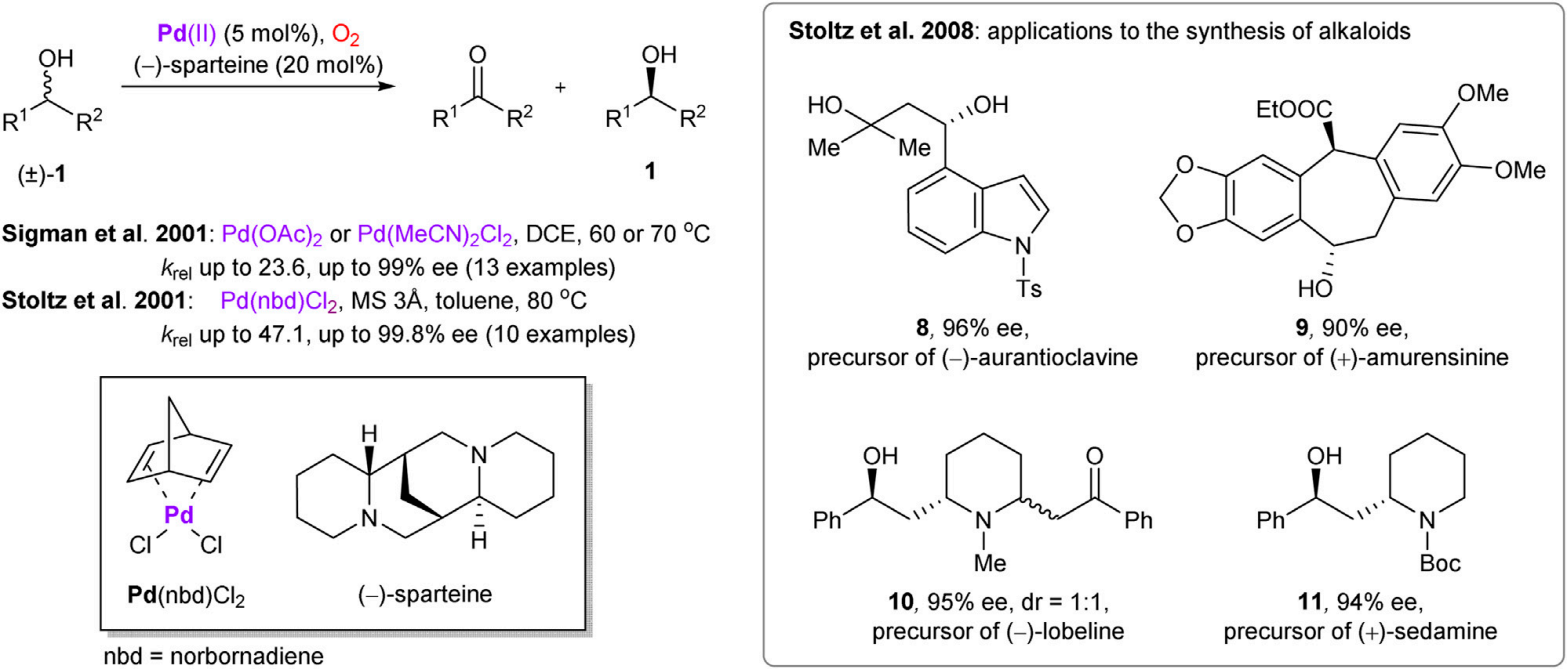
Palladium-catalyzed aerobic oxidative kinetic resolution of secondary alcohols and its application in the total synthesis of alkaloids (Ferreira and Stoltz, 2001; Jensen et al., 2001; Caspi et al., 2004; Krishnan et al., 2008).

Palladium is one of the most versatile and prominent transition metals in catalysis, including its well-documented role in aerobic oxidative transformations (Stahl, 2004). In 2001, the enantioselective palladium-catalyzed oxidation of secondary alcohols with molecular oxygen was independently reported by Stoltz’s (Ferreira and Stoltz, 2001) and Sigman’s groups (Jensen et al., 2001) (Scheme 3). The chirality was enabled by (–)-sparteine ligand, which also acted as a base. Later, Stoltz and co-workers presented application of this methodology to obtain pharmaceutically potent building blocks (Caspi et al., 2004) and in enantioselective synthesis of several alkaloids (Scheme 3) (Krishnan et al., 2008). The precursors **8–11** of the targeted natural products were prepared with exceptional enantioselectivity (90–96% ee) and respectable yields (compared to 50% theoretical maximum), enabling facile preparation of the required chiral motifs. Noteworthy, several functional groups within the substrates (e.g., free hydroxyls, esters) were compatible with the reaction conditions.

In recent decades, investigation of the catalytic reactions with earth-abundant transition metals as sustainable alternatives to precious metal catalysts has become a general trend. Several chiral complexes of the first-row transition metals have been applied in the catalytic oxidative kinetic resolutions of secondary alcohols with molecular oxygen as an oxidant. In these transformations, transition metals acted as oxygen carriers and/or chiral Lewis acids to enable stereodifferentiation of racemic substrates.

**SCHEME 4:**
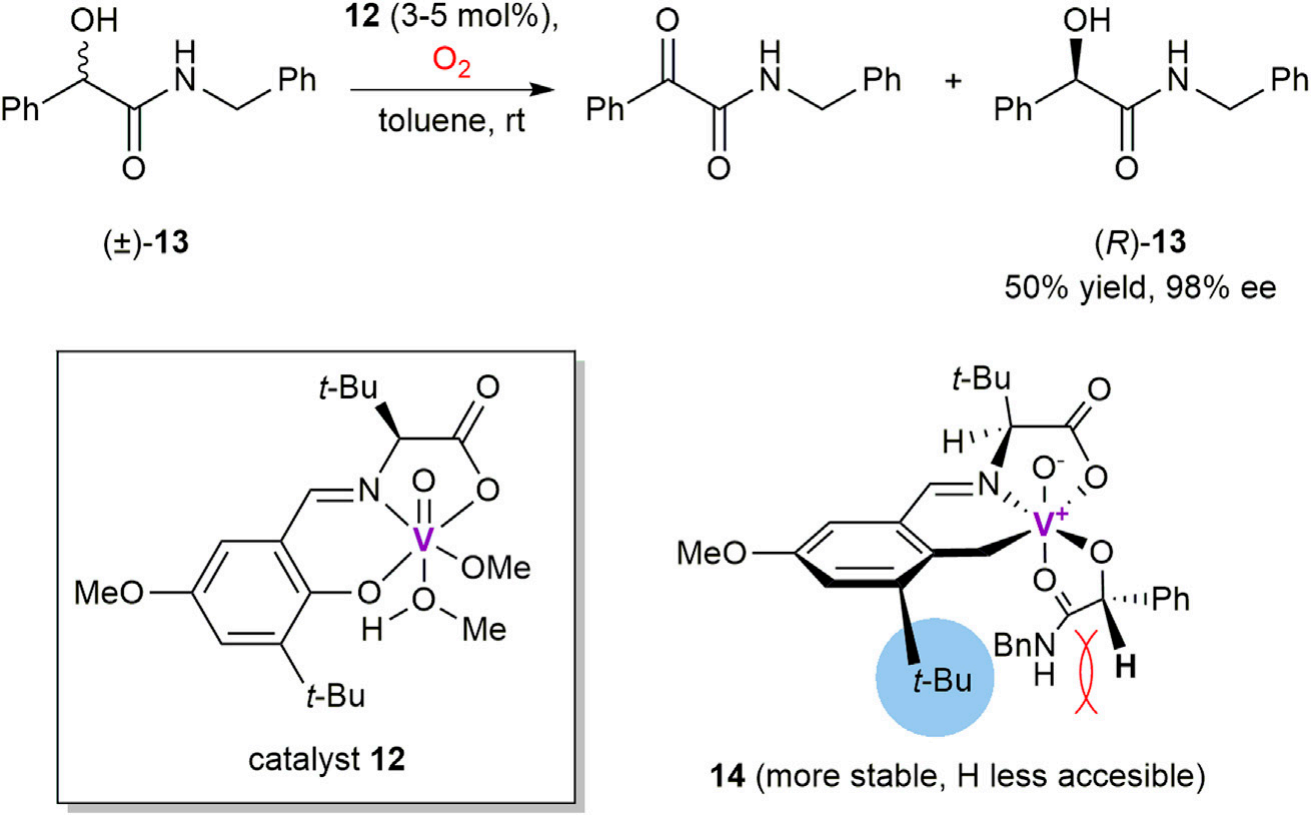
Kinetic resolution of *N*-benzyl mandelamide **13** via vanadium-mediated aerobic oxidation (Weng et al., 2006).

Thus, Chen et al. developed a series of chiral *N*-salicylidene vanadyl carboxylate complexes to perform catalytic kinetic resolution of α-hydroxycarboxylic esters, thioesters, amides (Weng et al., 2006; Chen et al., 2007; Salunke et al., 2011) and α-hydroxyketones (Chen et al., 2011) by using molecular oxygen as the terminal oxidant at ambient conditions. It was found that aerobic oxidation of (*S*)-alcohols proceeds more readily than their (*R*)-counterparts in the presence of vanadium complex **12** and its congeners (Scheme 4). As a notable example illustrating high oxidation selectivity, (*R*)-isomer of *N*-benzylamide **13** was obtained in 50% yield and 98% ee (Weng et al., 2006). Drastically slower rate of the oxidation for (*R*)-isomer (*k*_rel_ = 458) was rationalized based on the results of single crystal X-ray analysis of the corresponding catalyst-substrate adduct **14**. It was found that α-carbinol hydrogen of (*R*)-**13** is shielded with a neighboring *tert*-butyl group of the chiral ligand, thus making it barely accessible for elimination through a two-electron oxidation process (Weng et al., 2006). However, the shielding is absent in the diastereomeric complex with (*S*)-**13**, what results in its fast transformation into the α-ketoamide oxidation product and low-valent vanadium complex. Reoxidation of the latter with molecular oxygen recovers the reactive vanadium (V) species. As a further development, reusable polystyrene-supported vanadium catalysts have also been prepared to promote oxidations of α-hydroxy (thio)esters and amides with enantioselectivities of up to 99% ee (Salunke et al., 2011).

**SCHEME 5:**
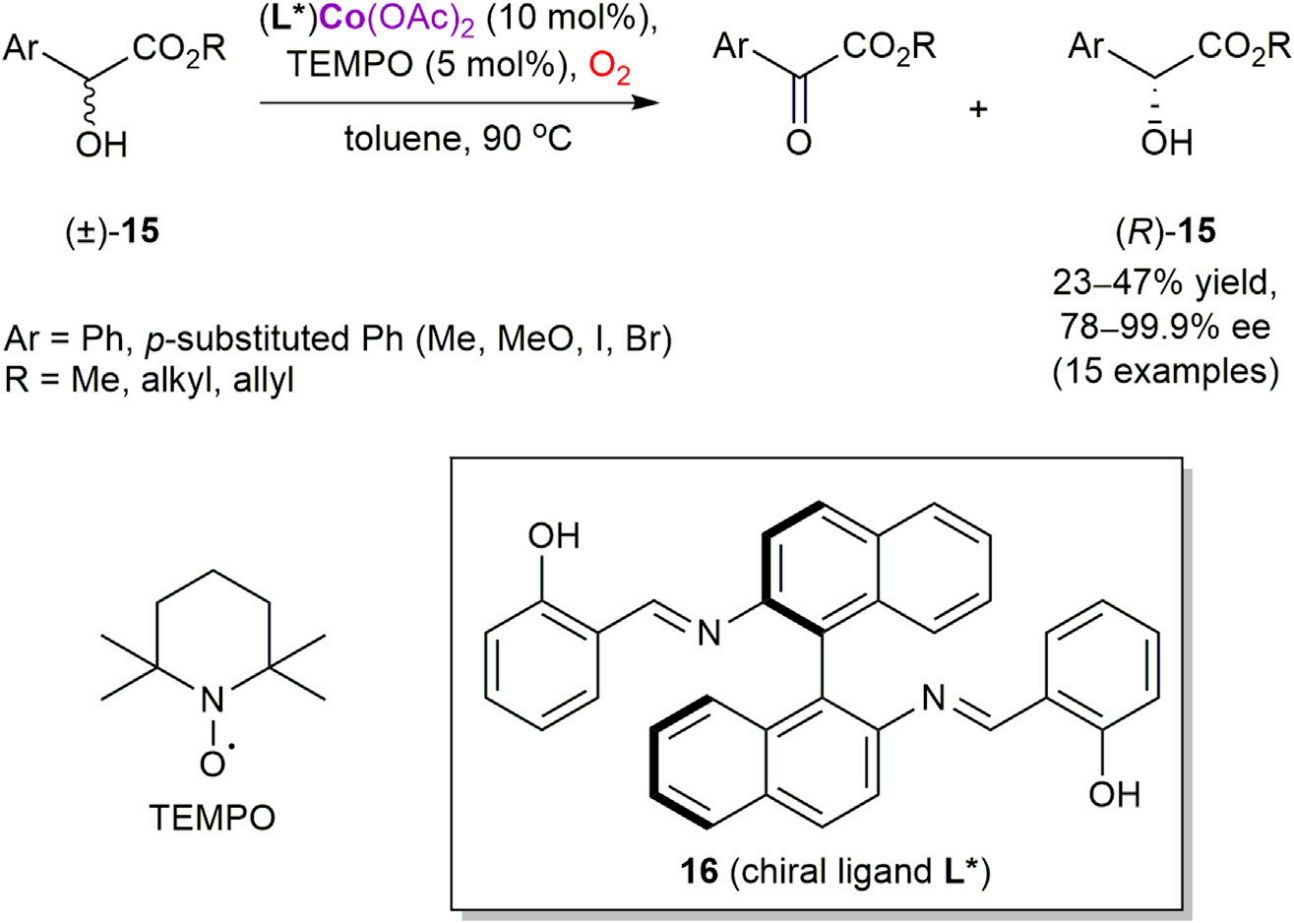
Cobalt-catalyzed enantioselective aerobic oxidation of α-hydroxy esters (Alamsetti and Sekar, 2010).

Alamsetti and Sekar reported the first cobalt-catalyzed oxidative kinetic resolution of α-hydroxyesters **15** with molecular oxygen as the sole oxidant (Scheme 5) (Alamsetti and Sekar, 2010). However, 2,2,6,6-tetramethylpiperidin-1-oxyl (TEMPO) was required as a co-catalyst (Wertz and Studer, 2013). Among several cobalt salts and chiral ligands screened, Co(OAc)_2_ and salen ligand **16** provided the best reaction outcome with racemic methyl mandelate **15** (R = Me, Ar = Ph). The developed conditions were also suitable for a range of α-hydroxyesters, with the maximum selectivity of 99.9% ee achieved for allyl mandelate. Although no mechanistic studies have been performed and a role of cobalt as a potent oxygen carrier has not been manifested, coordination of **15** to the chiral metal complex producing diastereomeric complexes with the different rates of TEMPO-mediated aerobic oxidation could be suggested as a plausible scenario.

**SCHEME 6:**
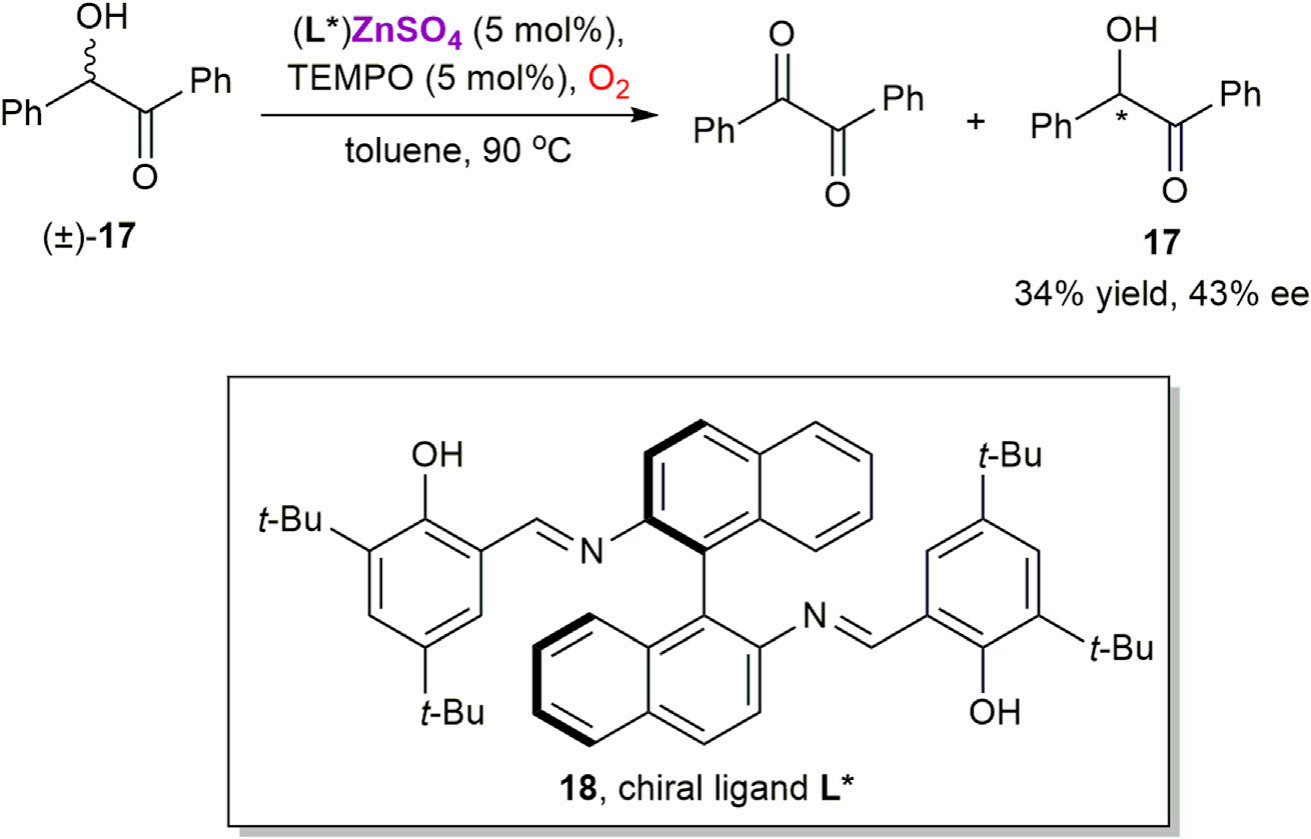
Oxidative kinetic resolution of (±)-benzoin **17** using chiral zinc catalyst (Muthupandi and Sekar, 2011).

The same group applied similar methodology for the kinetic resolution of benzoin **17** via the TEMPO-catalyzed aerobic oxidation in the presence of a chiral zinc complex, generated *in situ* from ZnSO_4_ and salen ligand **18** (Scheme 6) (Muthupandi and Sekar, 2011). This approach yields enantiomerically enriched **17** with low 43% ee. Since zinc possesses a single stable oxidation state +2 in its compounds, complex **17** could only play a role of chiral Lewis acid, while the oxidation should occur as a nitroxide-catalyzed process (Wertz and Studer, 2013).

**SCHEME 7:**
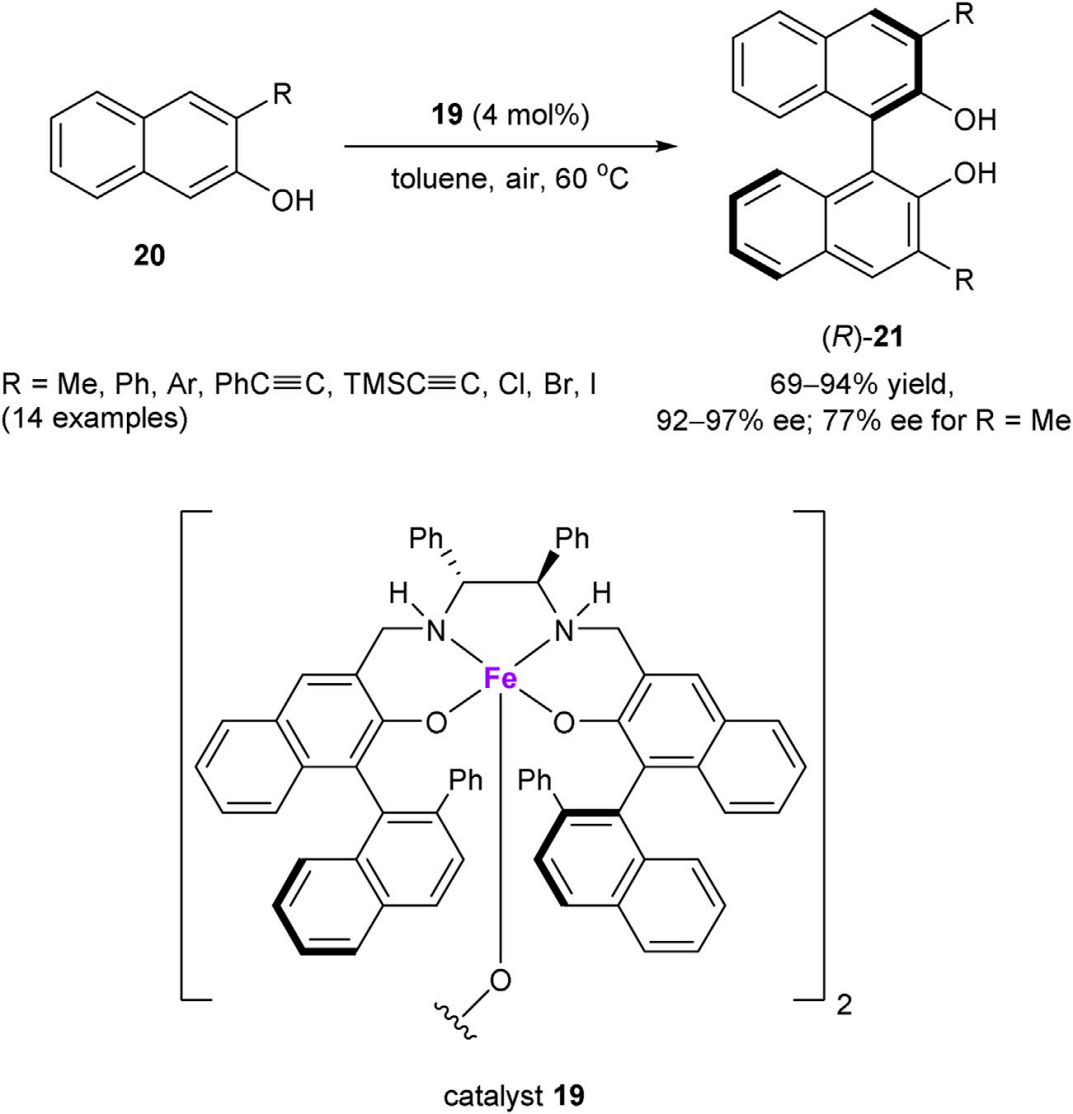
Iron-catalyzed aerobic oxidative coupling of 2-naphtols (Egami and Katsuki, 2009).

Dioxygen has also been utilized as an oxidant in the asymmetric dehydrogenative C–C coupling reactions. A notable example of such transformation was developed by Egami and Katsuki, who reported in 2009 an aerobic oxidative coupling of 2-naphthols mediated by Fe(salen) complexes (Scheme 7) (Egami and Katsuki, 2009). Chiral iron(III) complex **19** was found to be especially effective for the asymmetric coupling of 3-substituted 2-naphthols **20**, featuring bi-2-naphthols (*R*)-**21** with enantioselectivities greater than 90% ee, with the only exception of methyl-substituted compound (R = Me, 77% ee). The mechanistic studies suggested the coordination of a naphthol substrate to Fe^III^ in a chiral complex, followed by the “oxidase mode” SET oxidation with dioxygen as a rate-determining step. This process generates the radical cationic naphthol species, undergoing subsequent cross-coupling with the anionic 2-naphthol counterpart (Matsumoto et al., 2012; Uchida and Katsuki, 2013).

**SCHEME 8:**
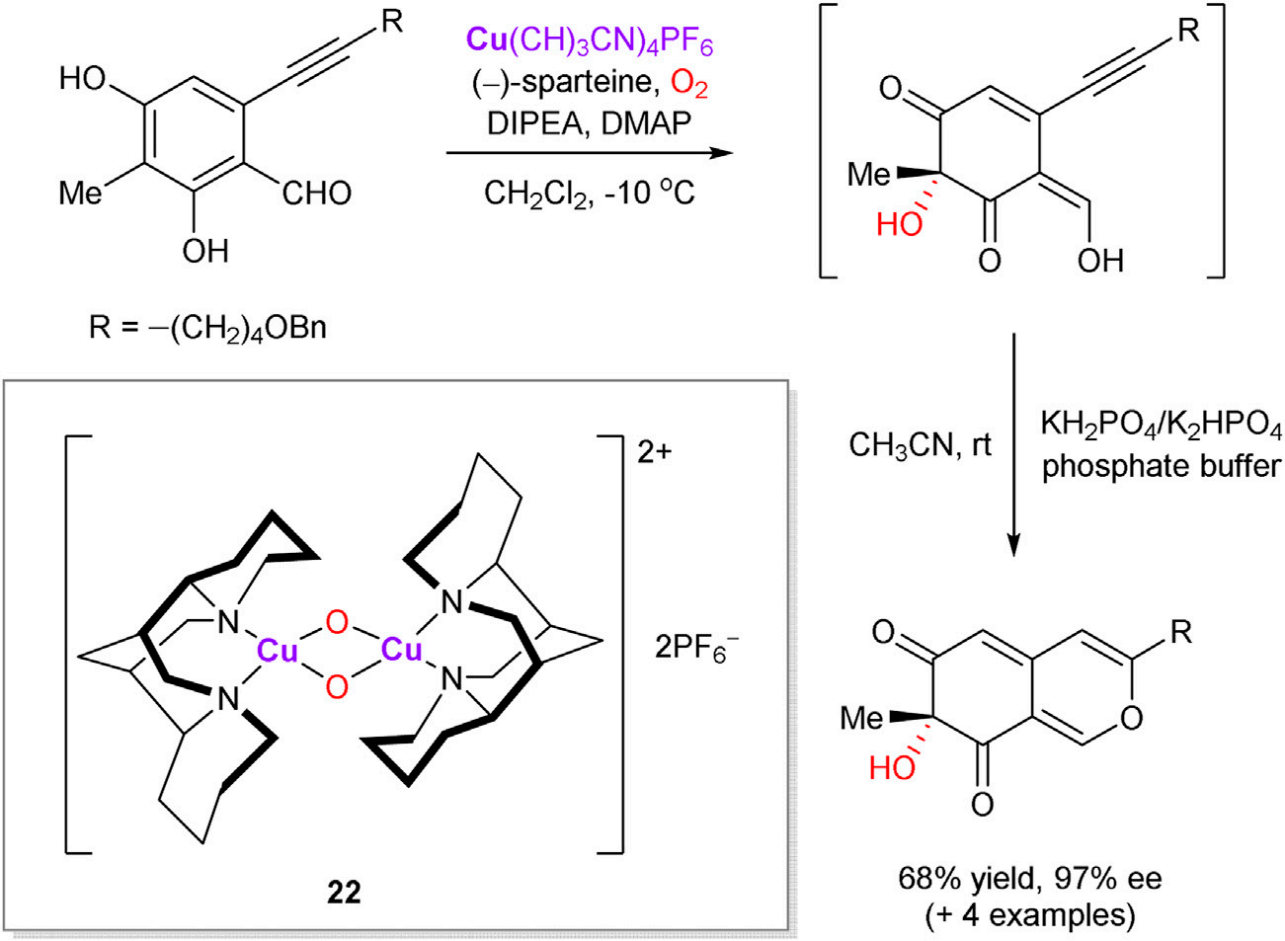
Copper-mediated enantioselective oxidative dearomatization of phenols (Zhu et al., 2005).

Chiral binuclear copper oxo-complex **22**, generated *in situ* from (–)-sparteine, Cu(CH_3_CN)_4_PF_6_ and molecular oxygen, was introduced by the Porco group to perform asymmetric dearomatization of phenolic compounds (representative example is shown in Scheme 8). The approach was successfully applied as a key step in asymmetric synthesis of natural products, such as azaphilones (Zhu et al., 2005; Zhu and Porco, 2006; Qian et al., 2007; Germain et al., 2011).

**SCHEME 9:**
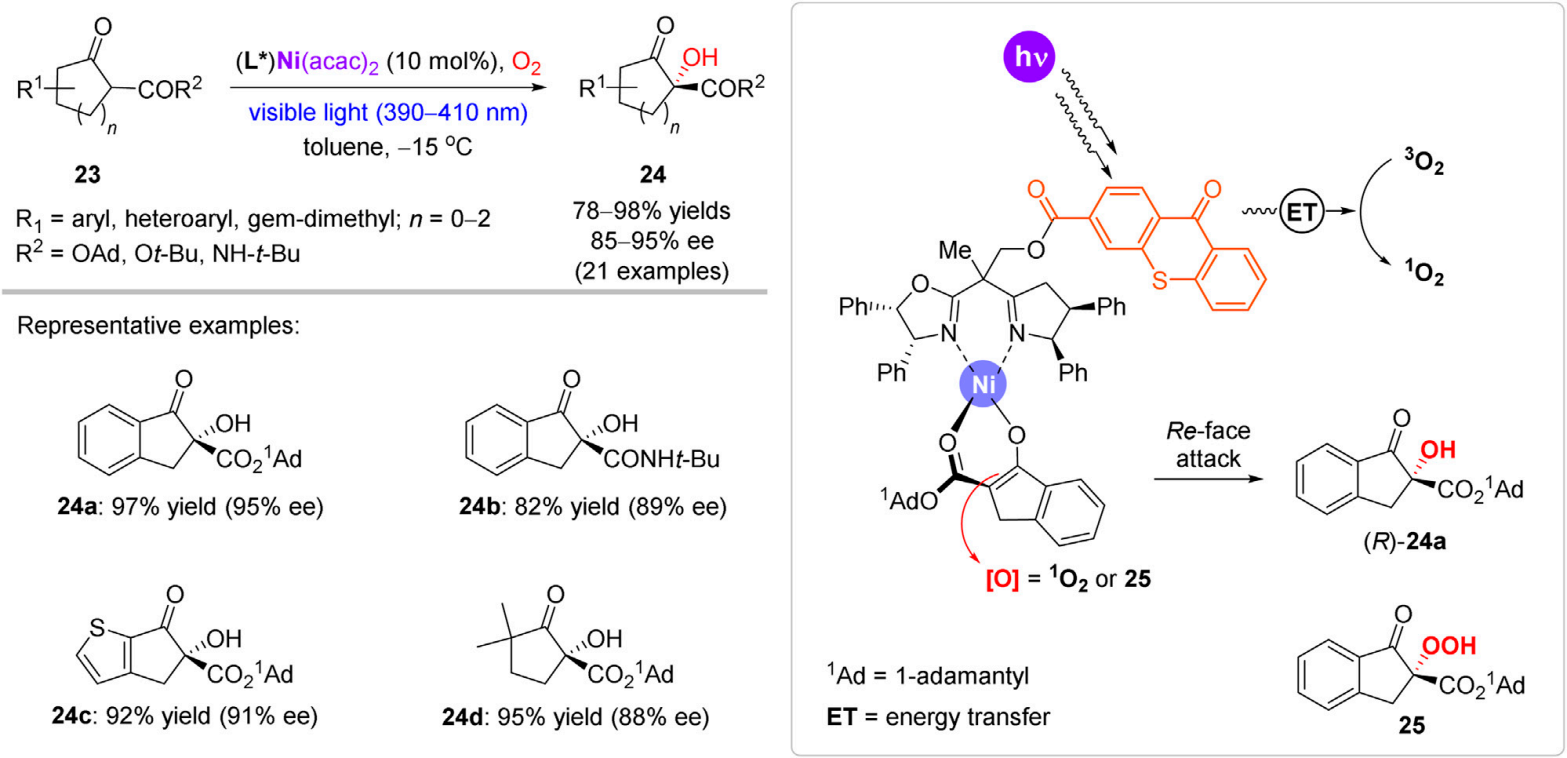
Enantioselective aerobic oxidation of β-ketoesters mediated by a chiral nickel complex (Ding et al., 2017).

In contrast to the mainstream use of transition metals as oxygen carriers or redox catalysts, Xiao et al. developed a synergetic transition metal-photocatalytic approach, which was applied for highly enantioselective α-hydroxylation of β-ketoesters and β-ketoamides **23** (Scheme 9) (Ding et al., 2017). A family of visible-light-responsive chiral ligands was prepared by grafting a photosensitive thioxanthone motif onto a chiral bisoxazoline scaffold. In the catalytic complex, the thioxanthone motif acts as a triplet-state sensitizer to enable photogeneration of singlet oxygen, while the Ni^2+^ cation acts as a Lewis acid to coordinate β-keto ester substrate **23** in the enolate form. Besides the preparation of indanone-derived α-hydroxylated esters and amides (e.g. **24a** and **24b**), the reaction protocol was also suitable for α-hydroxylation of heterocyclic and even aliphatic substrates (e.g. **24c** and **24d**). Several functional groups prone to oxidation (alkynyl, vinyl, thiophene, etc.) were inert under the reaction conditions. Asymmetric induction model was proposed, assuming the most preferable coordination of **23** with the most distant position of the bulky adamantyl group (^1^Ad) from the chiral ligand. Such mode of the coordination enables the *Re*-face attack of oxidants (either activated ^1^O_2_ or peroxide **25**), since the *Si*-face is blocked by the rear phenyl groups of the ligand. The model agrees with the experimentally assigned (*R*)-configuration of product **24a**.

### Dual Catalytic Transformations With a Complementary Asymmetric Step

Another important group of synthetic methods is based on the generation of a prochiral oxidized intermediate, which is subsequently involved into a complementary asymmetric catalytic cycle (Figure 3). Commonly, but not necessarily, the first oxidative catalytic step is transition metal-mediated, while the second asymmetric step is organocatalytic.

**SCHEME 10:**
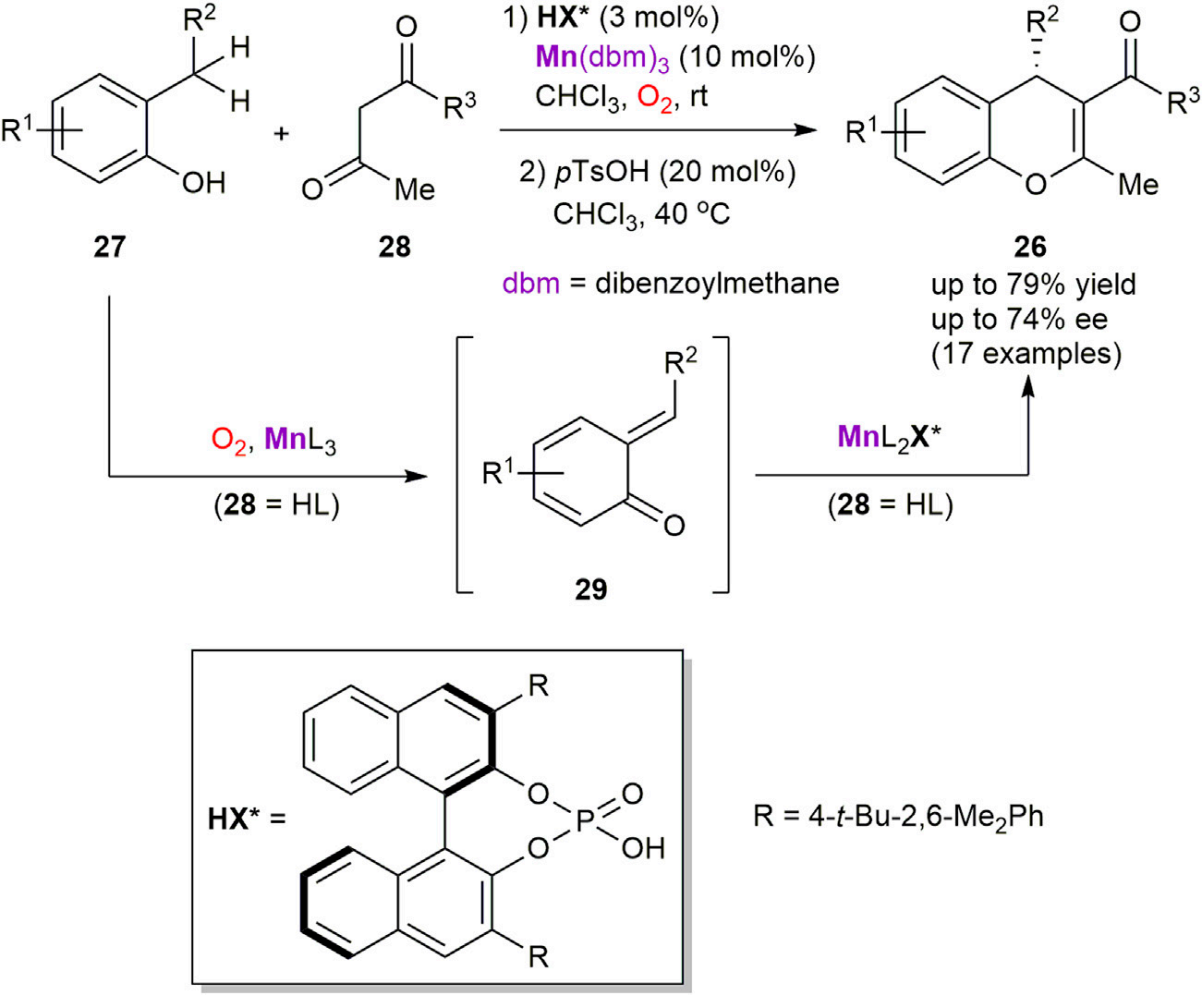
Asymmetric synthesis of 4*H*-chromenes (Gebauer et al., 2017).

Hence, the enantioselective synthesis of 4*H*-chromenes **26** from 2-alkyl-substituted phenols **27** and β-diketones **28** was developed by the Schneider group (Scheme 10) (Gebauer et al., 2017). The process consists of two stages and features a relay catalysis approach with two *in situ* generated manganese catalysts. In particular, the corresponding β-diketonato complexes of Mn^III^, generated from Mn(dbm)_3_ pre-catalyst and β-diketones **28**, were identified as superior oxygen carriers to perform *in situ* generation of transient ortho-quinone methides **29** by oxidation of phenols **27**. At the second stage, asymmetric Michael addition of **28** to **29** was occurred and mediated by another type of the chiral Mn^III^ complexes, generated from Mn^III^ diketonates and enantiopure phosphoric acid HX*. The process furnished 4*H*-chromenes **26** in up to 79% yield and 74% ee. However, the reaction outcome was found to be very sensitive to structural changes in the substrates **27** and **28**.

**SCHEME 11:**
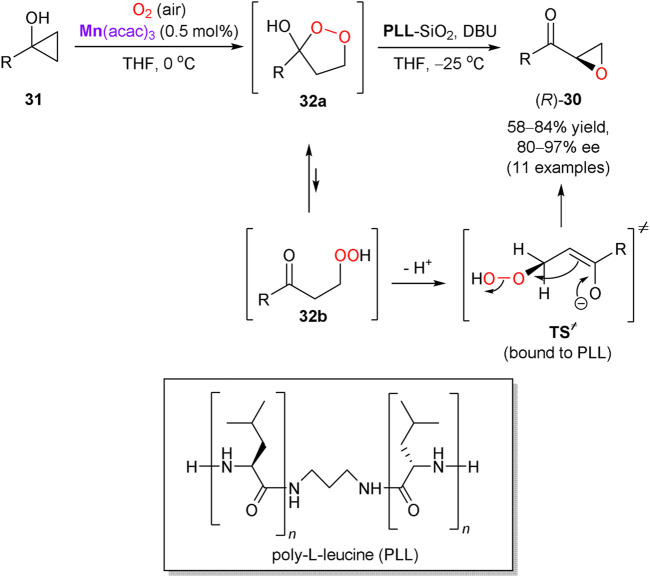
Asymmetric synthesis of epoxy ketones via aerobic oxidation of cyclopropanols (Elek et al., 2017).

In another representative example, Kananovich’s group developed a two-step and one-pot protocol for the asymmetric synthesis of epoxy ketones **30** from easily available tertiary cyclopropanols **31** (Scheme 11) (Elek et al., 2017). Oxidation of **31** with atmospheric oxygen proceeded readily in THF in the presence of manganese(III) acetylacetonate catalyst, to afford prochiral 1,2-dioxolanes **32a** (equilibrating with β-peroxo ketones **32b**) in nearly quantitative yields. The intermediates **32** were further converted into chiral epoxy ketones **30** by treatment with 1,8-diazabicyclo[5.4.0]undec-7-ene (DBU) in the presence of immobilized on silica gel poly-L-leucine (PLL) catalyst. The experimental protocol is operationally simple and affords **30** in generally high yields and enantioselectivities (80–97% ee). Several functionalities in the R group of **31** tolerate the reaction conditions, except of the metal-coordinating (e.g. amine) and base-sensitive moieties. The α-helical structure of PLL catalyst is responsible for the supramolecular binding of prochiral substrate **32** and favors the corresponding transition state (**TS** ^**≠**^) leading to the *R*-enantiomer of **30** (Kelly and Roberts, 2004; Berkessel et al., 2006). The developed transformation was successfully applied in a short and stereodivergent synthesis of chlamydocin, a natural histone deacetylase inhibitor with chiral epoxy ketone warhead (Elek et al., 2019).

**SCHEME 12:**
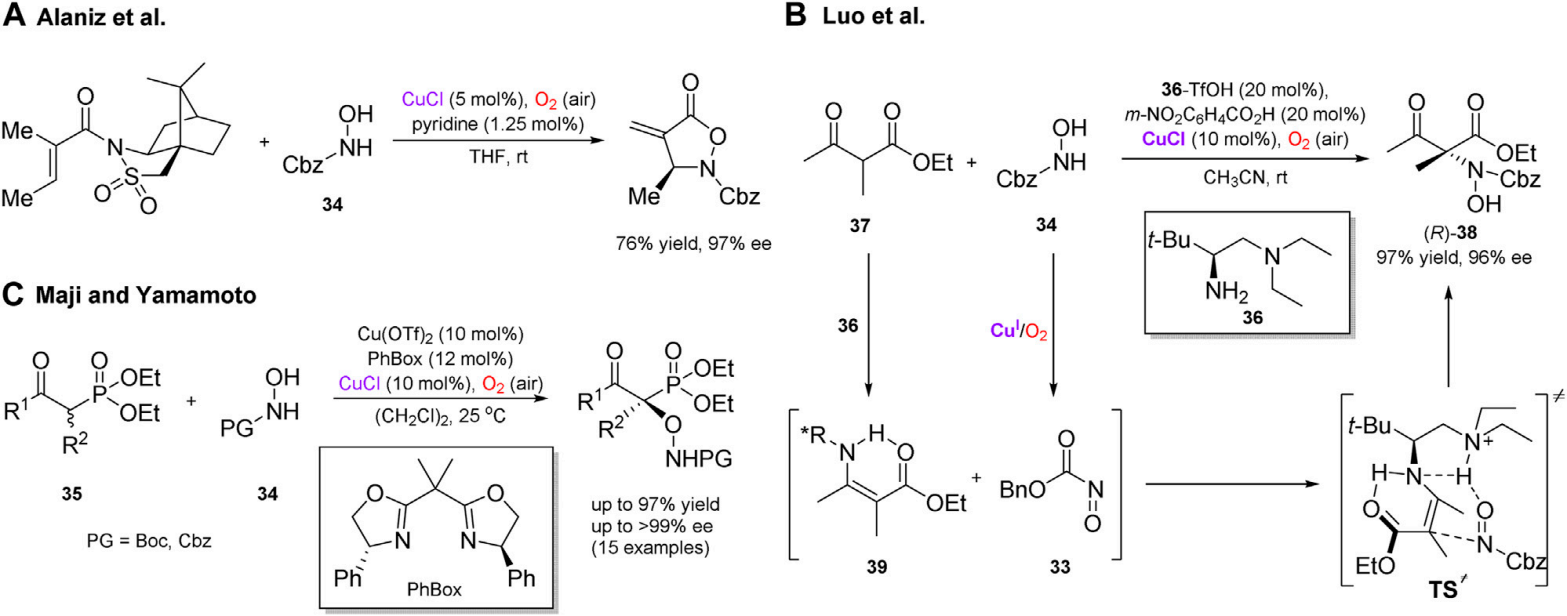
Versatile asymmetric transformations with nitroso compounds generated *in situ* by aerobic oxidation (Frazier et al., 2011; Xu et al., 2014; Maji and Yamamoto, 2015).

Several enantioselective cascade transformations with reactive intermediates generated by aerobic oxidation in the presence of copper salts have also been reported. Thus, Whiting (Chaiyaveij et al., 2011) and Alaniz (Frazier et al., 2011; Frazier et al., 2012) independently disclosed a convenient protocol for the generation of nitrosocarbonyl compounds (e.g. **33**, Scheme 12B) by copper-catalyzed aerobic oxidation of *N*-protected hydroxylamines **34**. These reactive electrophilic nitroso compounds have been utilized in a number of robust transformations, including the asymmetric ene reactions (Scheme 12A), (Frazier et al., 2011) inter- and intramolecular hetero-Diels–Alder reactions, (Chaiyaveij et al., 2011; Frazier et al., 2012) α-amination of silyl enol ethers, (Sandoval et al., 2015) β-dicarbonyl compounds (Scheme 12B), (Xu et al., 2014) and α-hydroxylation of β-ketophosphonates **35** (Scheme 12C) (Maji and Yamamoto, 2014; Maji and Yamamoto, 2015). The asymmetric α-amination of β-ketocarbonyl compounds under aerobic conditions, developed by Luo et al., can represent a notable example (Scheme 12B) (Xu et al., 2014). In this transformation, aerobic generation of nitrosocarbonyls was beneficially merged with enamine catalysis enabled by a chiral primary amine **36**. The process featured high chemo- and enantioselectivity for a broad range of β-ketocarbonyl substrates, for example β-ketoester **37** furnished the corresponding amination adduct (*R*)-**38** in 97% yield and 96% ee. Based on the absolute configuration of product **38**, as well as X-ray crystal and solution structures of enamine intermediate **39**, the transition state was suggested to account for the observed stereoselectivity, in which the hydrogen-bonding network between **39** and **33** facilitates the *Re*-face attack of enamine to give adduct **38** with the (*R*)-configuration.

**SCHEME 13:**
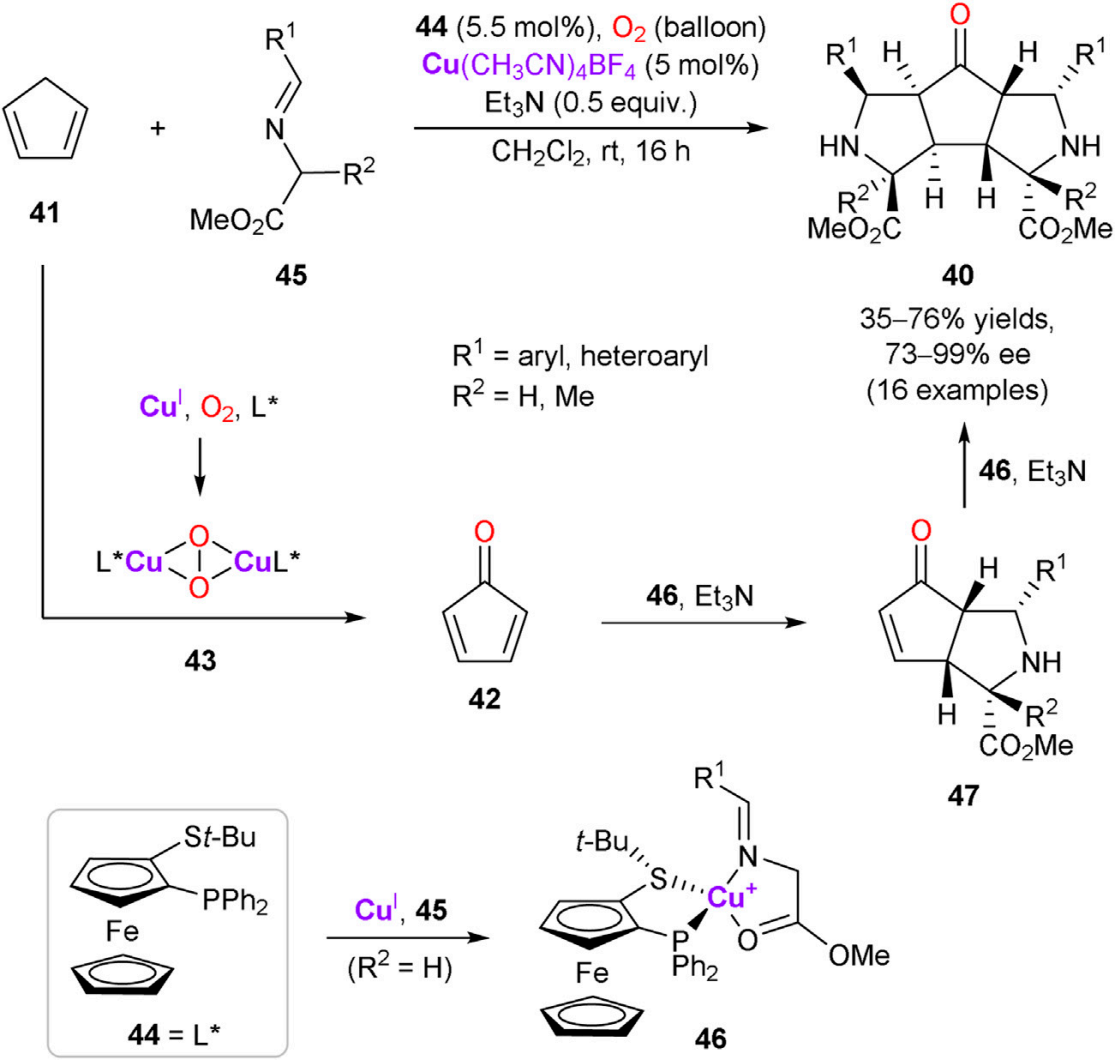
Catalytic aerobic oxidation of cyclopentadiene and tandem enantioselective cycloaddition (Potowski et al., 2015).

An interesting example of the enantioselective assembly of structurally complex 5,5,5-tricyclic products **40** with eight stereocenters via the multicomponent cascade reaction was described (Scheme 13) (Potowski et al., 2015). The cascade process is triggered by copper-catalyzed aerobic oxidation of cyclopentadiene **41** to cyclopentadienone **42**. It was supposed that the allylic C–H oxidation of **41** is mediated by the copper peroxo-complex **43**, formed by trapping molecular oxygen by Cu(CH_3_CN)_4_BF_4_ in the presence of (*R*)-Fesulphos ligand **44**. The subsequent double catalytic asymmetric 1,3-dipolar cycloaddition of azomethine ylides, derived from glycine ester imines **45**, delivers the final tricyclic product **40**. In turn, azomethine ylides are generated by deprotonation of the glycine ester imine ligand in the chiral copper complex **46**. The step-wise double cycloaddition proceeds via the *endo* transition state with **42** (or intermediate **47**) approaching from the *Re*-face of ylide (with respect to C=N) to avoid unfavorable interactions with the *t*-Bu group of the ligand **44**.

**SCHEME 14:**
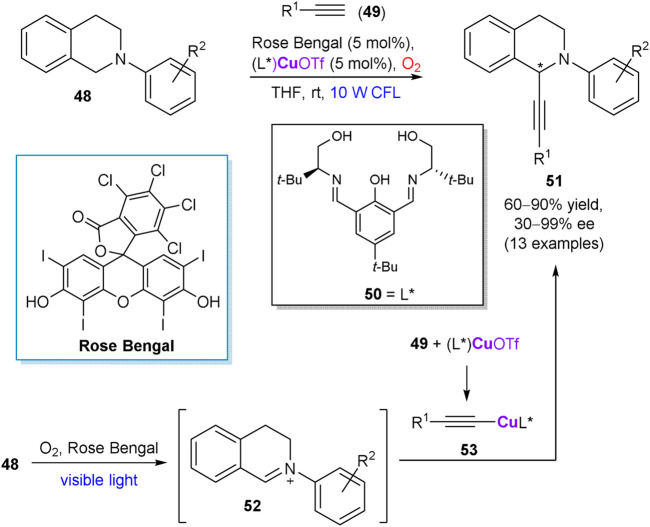
Enantioselective cross dehydrogenative coupling of alkynes **49** with tetrahydroisoquinoline derivatives **48** (Kumar et al., 2017).

In 2017, Khan et al. described a visible-light enabled enantioselective cross dehydrogenative coupling between tetrahydroisoquinoline derivatives **48** and alkynes **49**, using molecular oxygen as the sole oxidant (Scheme 14) (Kumar et al., 2017). The reaction was mediated by *in situ* generated copper complex with chiral salen ligand **50** and Rose Bengal as a complementary photo-redox catalyst, furnishing alkynylation products **51** in up to 90% yield and up to 99% ee. According to the suggested reaction mechanism, prochiral iminium ion **52** is produced by oxidation of **48** in the photo-redox catalytic cycle. In turn, the chiral copper complex (L*)CuOTf activates terminal alkyne **49** and serves as a precursor of chiral acetylide species **53**, which affords the optically active coupling product **51** after addition to the intermediate imine **52**.

**SCHEME 15:**
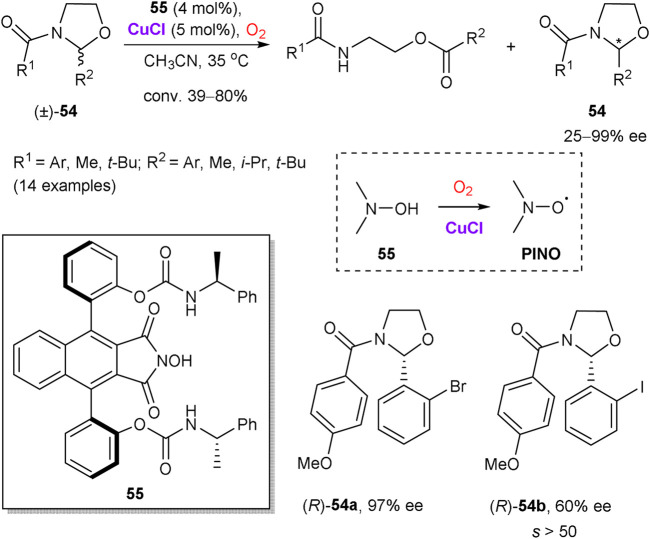
Copper-catalyzed oxidative kinetic resolution of oxazolidines **54** (Nechab et al., 2007).

Copper salts are also able to assist in the recovery of catalytically active nitroxide radical species by oxidation with molecular oxygen. Hence, Einhorn et al. disclosed kinetic resolution of oxazolidines **54** by the oxidative ring-opening, catalyzed by chiral *N*-hydroxyphthalimides (NHPI) and copper(I) chloride (Scheme 15) (Nechab et al., 2007). Fast reaction rates for the aerobic oxidation of **54** and selectivity factors *s* > 20 were observed for a range of the substrates with the most efficient chiral NHPI catalyst **55**. As notable examples, *o*-bromophenyl-substituted oxazolidine (*R*)-**54a** was obtained in 97% ee at 58% conversion, while *o*-iodophenyloxazolidine **54b** had the ee value of 60% at 39% conversion that corresponds to *s* > 50. The reaction proceeds via intermediate phthalimide *N*-oxyl radical (PINO), which enables a hydrogen atom transfer from the substrate **54**. While a precise role of copper is unclear, it could be attributed to the formation of μ-oxocopper(II) species by the reaction of CuCl with dioxygen, which are capable to generate PINO from **55** (Nechab et al., 2004).

**SCHEME 16:**
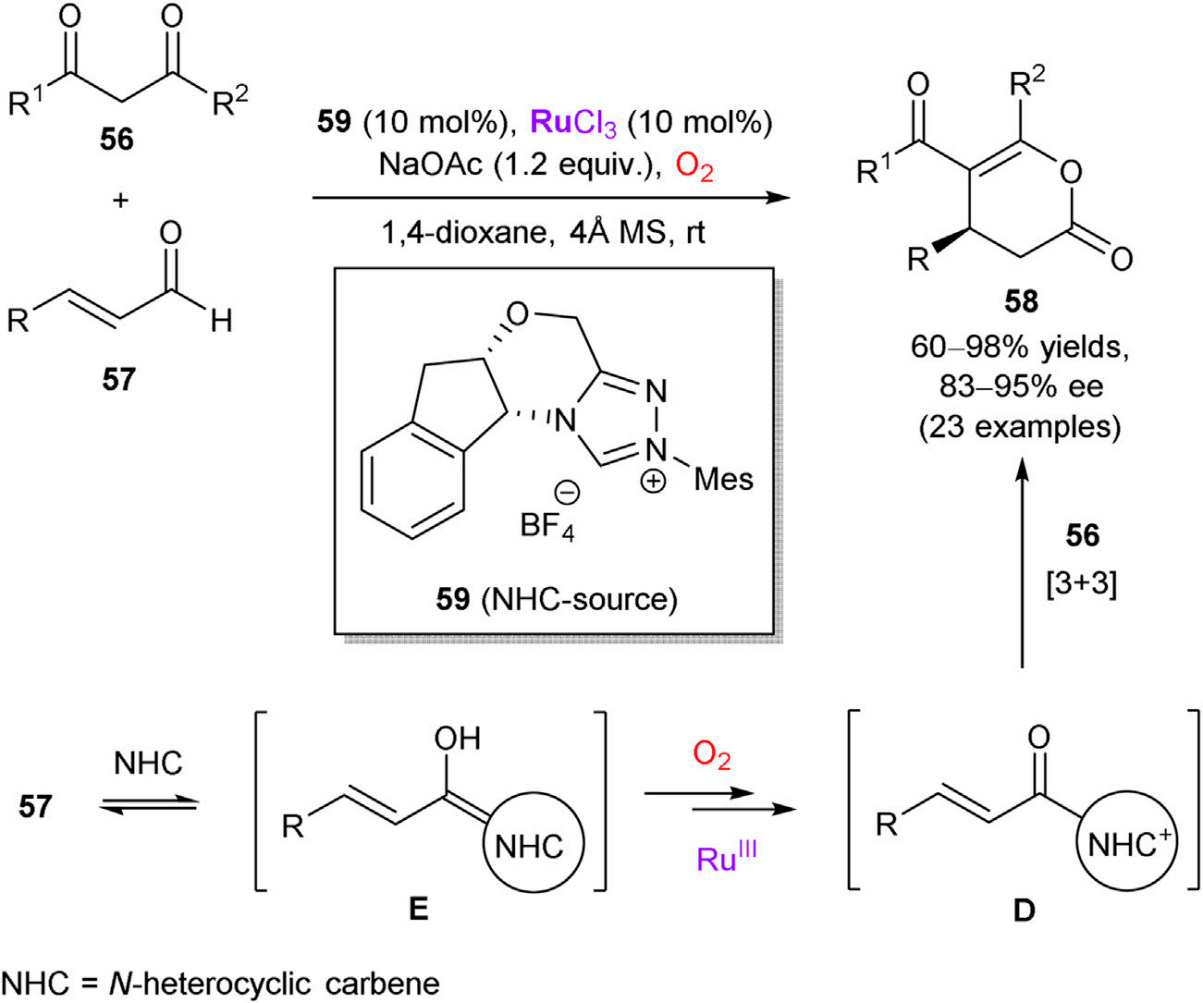
Aerobic oxidation/annulation cascade through synergistic catalysis of RuCl_3_ and *N*-heterocyclic carbene (NHC) precursor **59** (Wang et al., 2018).

The last representative example in this subtopic is based on the work of Wang et al., who demonstrated that coordinatively unsaturated RuCl_3_ can act as a synergistic co-catalyst in tandem with chiral *N*-heterocyclic carbenes (NHC) (Wang et al., 2018). The authors performed the aerobic [3 + 3] annulation reaction of β-dicarbonyl compounds **56** and enals **57** to access enantiomerically enriched lactones **58** in the presence of carbene precursor **59** (Scheme 16). The developed methodology exhibits a broad substrate scope, such as enals with complicated skeletons and diketones with exceptional structural diversity (23 examples in total). The obtained chemical yields were good-to-excellent along with the enantiomeric purity of up to 94% ee. According to the mechanistic studies, the reaction proceeds via acyl azolium intermediate **D**, generated by the ruthenium-catalyzed aerobic oxidation of homoenolate **E**. As a further extension, azolium dienolate intermediates can be generated from β,β-disubstituted enals, which smoothly afford δ-chiral lactones with 80–84% ee upon the [4 + 2] annulation reaction with trifluoromethyl ketones.

## Metal-Free Catalytic Systems

Another important synthetic strategy of asymmetric aerobic oxidations is based on the metal-free catalytic systems. The research activity in this field was commenced in the late 1980s by Shioiri, who described the first α-hydroxylation of achiral ketones with molecular oxygen in a two-phase system, which was mediated by chiral phase transfer catalysts (PTC) and triethyl phosphite as a co-reductant (Masui et al., 1988). In 1995, Brussee utilized chiral aza-crown ethers for the same purpose (de Vries et al., 1995). The chiral phase transfer catalysis still represent the most frequently exploited approach among the organocatalytic aerobic oxidation methods. Importantly, a specific dioxygen activation process may not be required, since many enolizable carbonyl compounds can readily react with molecular oxygen under basic conditions (Doering and Haines, 1954; Gardner et al., 1968; Konen et al., 1975). As a rule, the organocatalytic methods are tolerant to both air and water what makes them especially attractive for various practical applications.

**SCHEME 17:**
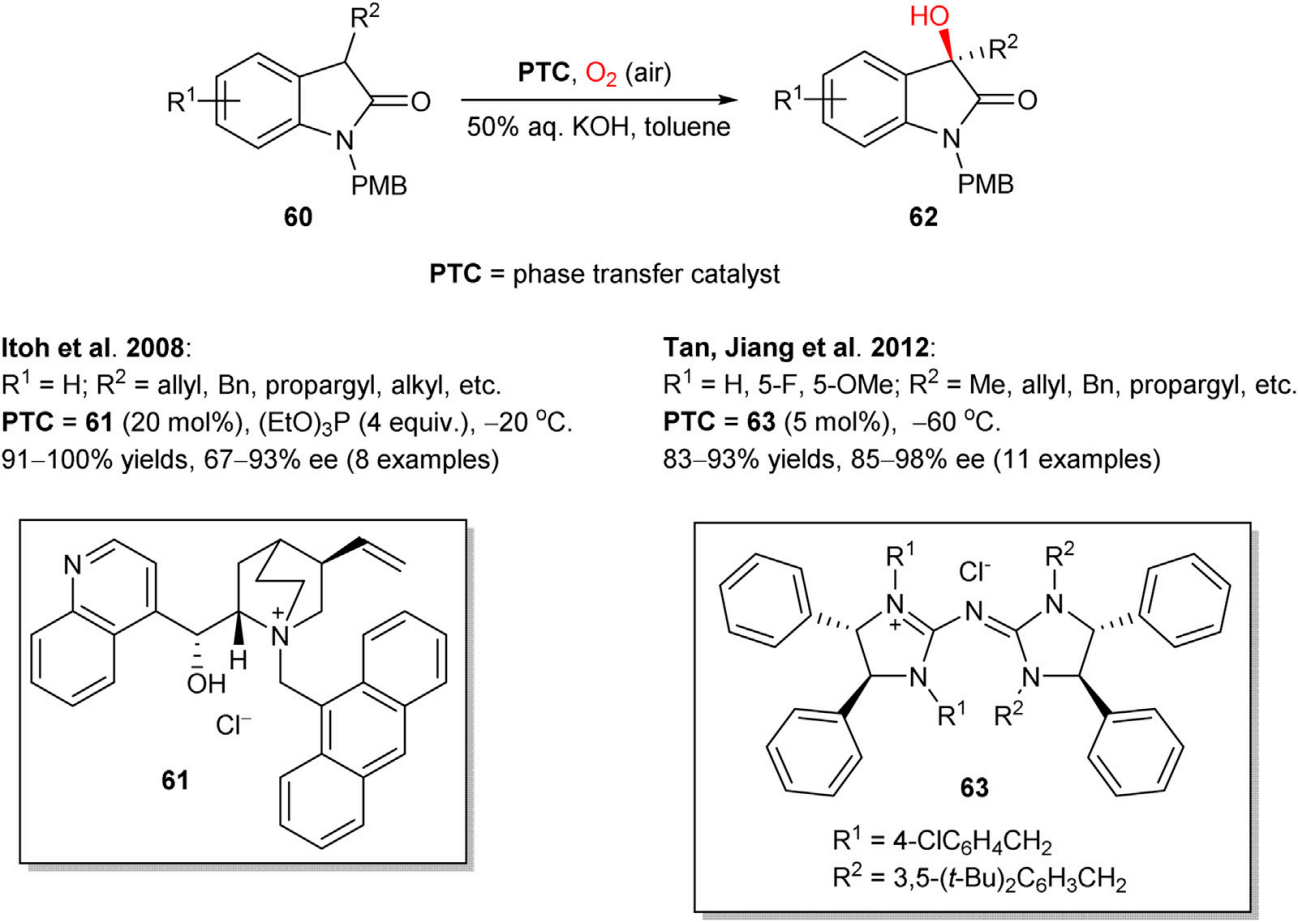
Asymmetric hydroxylation of oxindoles **60** under phase transfer catalysis (Sano et al., 2008; Yang et al., 2012).

**SCHEME 18:**
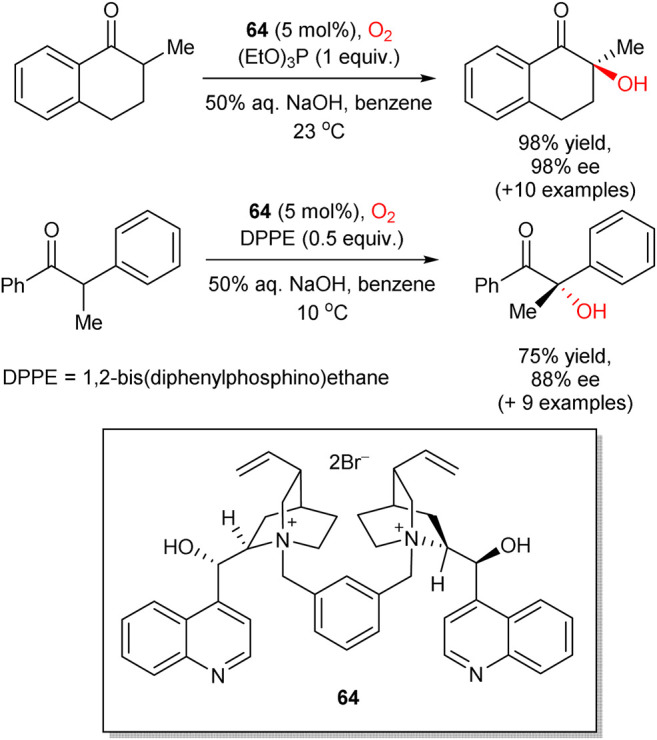
Enantioselective α-hydroxylation of cyclic and acyclic ketones (Sim et al., 2015).

Following the seminal works of Shioiri (Masui et al., 1988) and Brussee, (de Vries et al., 1995) in 2008, Itoh group reported asymmetric hydroxylation of oxindoles **60** in the biphasic solvent system (toluene/water), mediated by chiral cinchonidine-derived PTC **61** (Scheme 17) (Sano et al., 2008). In this transformation, a set of 3-alkyl-, alkenyl-, and alkynyl-substituted oxindoles **60** were hydroxylated by a simple reaction of oxindole-derived enolates with molecular oxygen and triethyl phosphite, acting as a reductant for peroxide intermediates. The process furnished the corresponding 3-hydroxylated oxindole products **62** in up to quantitative yields and 67–93% enantioselectivities. The (*R*)absolute configuration at the stereogenic center in **62** (for R = allyl) was confirmed by chemical correlation with the known indole compound. In 2012, Tan and Jiang designed similar transformation, utilizing pentanidinium PTC **63** (Yang et al., 2012). In comparison to the Itoh’s work, in this case a lower catalyst loading (5 mol%) was sufficient and no additional reductant such as triethyl phosphite was necessary. In 2015, Zhao et al. followed a similar phase-transfer catalytic strategy with 1,2-bis(diphenylphosphino)ethane or triethyl phosphite as reductants to perform the aerobic enantioselective α-hydroxylation of cyclic and acyclic ketones with higher yields and enantioselectivities achieved for cyclic ketones (representative examples are shown in Scheme 18). Dimeric PTC **64** was utilized and provided excellent stereoinduction at only 5 mol% loading (Sim et al., 2015).

**FIGURE 4:**
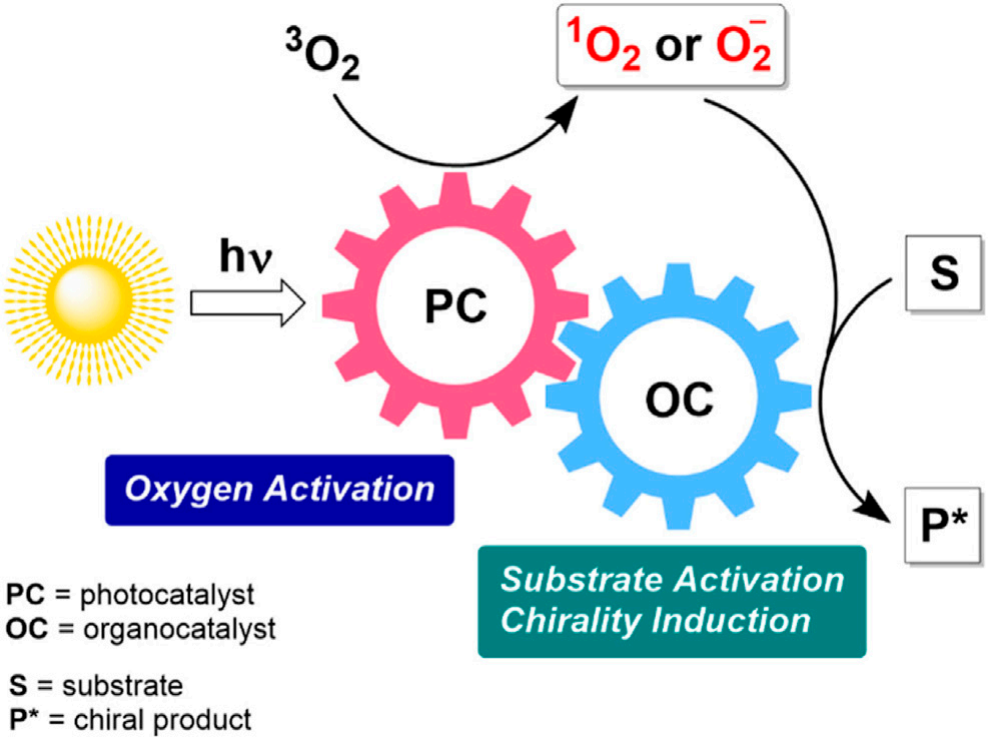
Schematic representation of tandem photo-organocatalysis in asymmetric metal-free aerobic oxidations.

Several recent developments relied on combined photo-organocatalysis to produce more reactive oxygen species, as schematically outlined in Figure 4. The photochemical activation of dioxygen delivers highly reactive singlet oxygen in the presence of organic dyes acting as photosensitizers. Alternatively, a photoredox catalytic cycle can be involved. In such a case, the generation of superoxide anion (O_2_^−^) in an oxidative quenching cycle can be considered as a plausible O_2_ activation pathway. Subsequent asymmetric induction and activation of a substrate take place in a complementary organocatalytic cycle, which may include different types of chiral organocatalytic species, e.g. secondary amines, hydrogen bonding catalysts, phase transfer catalysts. Notable, an organocatalytic molecule and an organic dye can be covalently linked to form a bifunctional catalyst.

**SCHEME 19:**
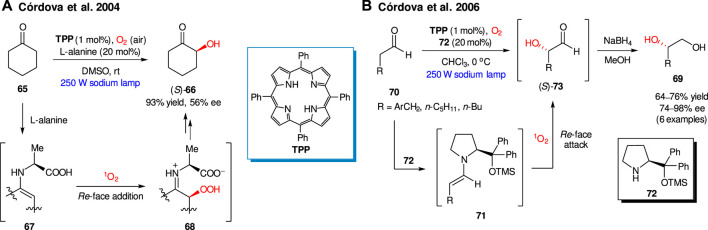
Enantioselective α-hydroxylations of carbonyl compounds with singlet oxygen (Sundén et al., 2004; Ibrahem et al., 2006).

The first example of photochemically-driven asymmetric organocatalytic oxidation with singlet oxygen was described by Córdova group, in 2004 (Sundén et al., 2004). They found that singlet oxygen, generated in the presence of tetraphenylporphyrin (TPP) as a photosensitizer, furnished the enantioselective α-oxidation of ketones in the presence of several natural amino acids as organocatalysts. For example, cyclohexanone **65** produced α-hydroxyketone (*S*)-**66** in 93% yield and 56% ee in the presence of L-alanine (20 mol%, Scheme 19A). The key steps of the proposed reaction mechanism imply the intermediate formation of enamine **67**, which undergoes the *Re*-face addition of singlet oxygen producing (2*S*)-α-hydroperoxide intermediate **68**. In the case of a cyclic L-amino acid (e.g. L-proline) the addition of ^1^O_2_ occurred to the *Si*-face of corresponding enamine, thus providing opposite (*R*)-enantiomer of **66**.

As further expansion of the method utility, in 2006 the Córdova group presented the synthesis of 1,2-diols **69** via organocatalytic enantioselective α-oxidation of aldehydes **70** (Scheme 19B) (Ibrahem et al., 2006). The process relies on the generation of enamine species **71**, formed *in situ* from aldehyde **70** and secondary amine organocatalyst **72**. The addition of ^1^O_2_ occurs from the more sterically accessible *Re*-face of enamine **71**. Reduction of produced α-hydroxy aldehydes **73** with NaBH_4_ furnished 1,2-diols **69** in 64–76% yields and 74–98% ee.

**SCHEME 20:**
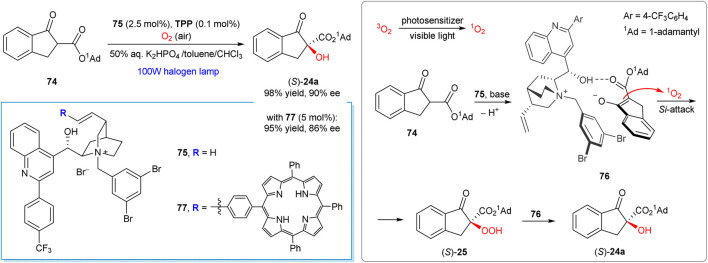
Enantioselective α-hydroxylation of β-keto esters with singlet oxygen under chiral phase transfer catalysis (Wang et al., 2016; Tang et al., 2018).

Works of the Meng group represent a notable recent example of chirality induction in the α-hydroxylation reactions by means of phase transfer catalysis and visible light activation of dioxygen (Lian et al., 2012; Wang et al., 2016; Tang et al., 2018; Tang et al., 2019a; Tang et al., 2019b). The method can be considered as complementary to the Ni-mediated transformation developed by the Xiao group (Scheme 9) (Ding et al., 2017). For example, the aerobic oxidation of ester **74** furnished hydroxylation product **24a** in 98% yield and 90% ee in the presence of 2.5 mol% of phase transfer catalyst **75** (Scheme 20) (Wang et al., 2016). The reaction conditions are mild, being applicable for a range of the β-carbonyl compounds (30 examples in total). Furthermore, the synthetic protocol is scalable and suitable for preparation of **24a** in gram quantities without the loss of enantioselectivity (Wang et al., 2016).

The key step of the suggested reaction mechanism (Scheme 20) implies the addition of singlet oxygen to the chiral enolate-PTC complex **76**, in which ion-paring, hydrogen bonding and π-π stacking between PTC and substrate were suggested as key supramolecular interactions (Wang et al., 2016). The produced hydroperoxide intermediate **25** then rapidly reacts with the enolate complex **76** furnishing the final product **24a**. The stereochemical outcome of the oxidation could be explained by the *Si*-face attack of ^1^O_2_ or hydroperoxide **25**, since the opposite *Re*-face of enolate in the complex **76** is shielded by 1-adamantyl group.

A bifunctional photo-organocatalyst **77** has also been developed by the same group, with a tetraphenylporphyrin unit grafted to the cinchona alkaloid-derived phase-transfer catalyst (Tang et al., 2018). With the catalyst **77** (5 mol%), visible-light induced aerobic oxidation of **74** afforded (*S*)-**24a** in 95% yield and 86% ee. Further methodological improvements, such as greatly reduced reaction times, were achieved by using a flow photomicroreactor technology (Tang et al., 2019a; Tang et al., 2019b).

Besides α-hydroxylation of carbonyl compounds, the asymmetric metal-free oxidations with molecular oxygen can also be applied for the synthesis of other valuable chiral products, although such examples are less common.

**SCHEME 21:**
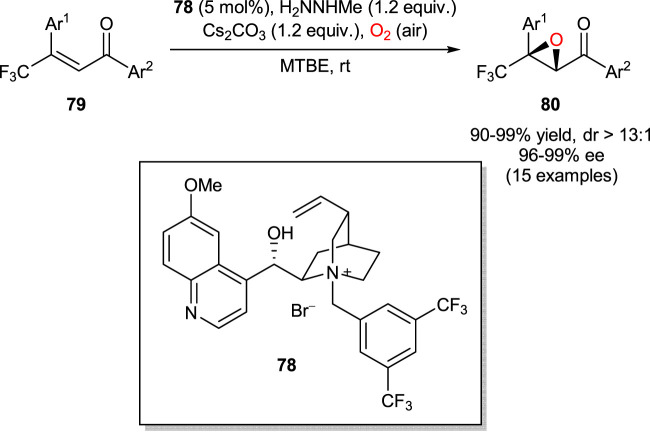
Enantioselective aerobic epoxidation of enones **79** (Kawai et al., 2013).

In 2013, Shibata group reported a highly enantioselective aerobic epoxidation of β-trifluoromethyl β,β-disubstituted enones in the presence of methylhydrazine and catalytic amounts (5 mol%) of cinchona alkaloid-derived PTC **78** (Scheme 21) (Kawai et al., 2013). The use of methylhydrazine was essential to enable a unique dioxygen activation pathway, in which highly pure hydrogen peroxide was produced *in situ* by reduction of molecular oxygen. The subsequent asymmetric Weitz-Scheffer reaction of enones **79** with H_2_O_2_ afforded chiral epoxide products **80**.

**SCHEME 22:**
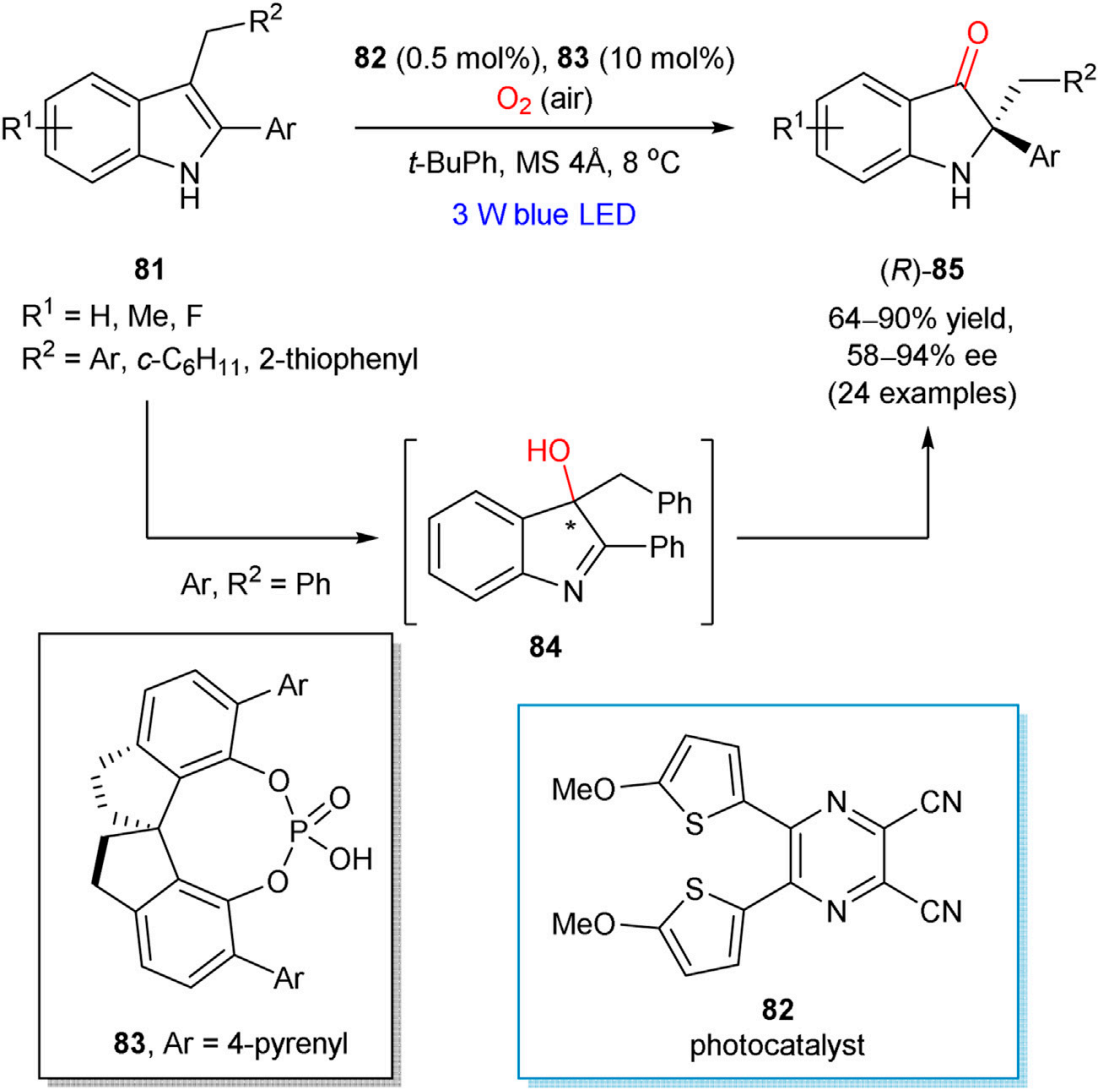
Enantioselective aerobic oxidation of 2-aryl-3-alkylsubstituted indoles **81** (Bu et al., 2018).

The enantioselective aerobic oxidation of 2-aryl-3-alkylsubstituted indoles **81**, accompanied with the semipinacol rearrangement, was reported by Zhao and Jiang in 2018 (Scheme 22) (Bu et al., 2018). The developed cooperative catalytic approach involved organophotoredox **82** and hydrogen bonding **83** catalysts. The preliminary mechanistic studies proved the formation of 3-hydroxylated compound **84** at the first step of aerobic oxidation. It was shown that chiral phosphoric acid **83** provided stereocontrol already at the first step, since the intermediate **84** was formed in its enantiomerically enriched form (59% ee at 65% conversion). However, higher enantioselectivity was observed in the subsequent pinacol rearrangement step (92% ee for **85**; Ar = R^2^ = Ph), indicating that the generated 3-hydroxy intermediate **84** could be engaged at this step and hence affected the enantioselectivity of this rearrangement. The reaction mechanism is apparently similar to that mediated by Ru(bpy)_3_Cl_2_ as a photocatalyst, which was thoroughly investigated by Xiao and Lu (Ding et al., 2014) and could involve the reaction between photochemically generated indole cation radical and superoxide radical anion, formed from molecular oxygen. While the generation of singlet oxygen cannot be completely excluded, it is unlikely a dominant pathway.

**SCHEME 23:**
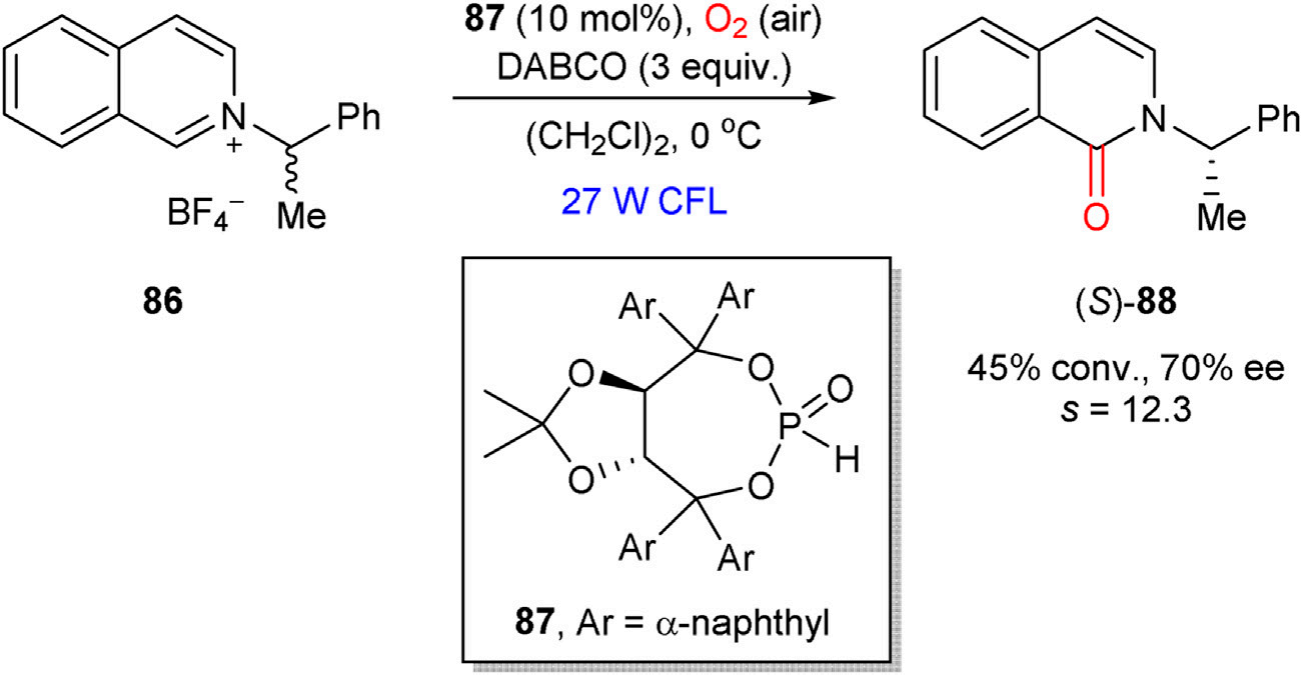
Kinetic resolution in aerobic oxidation of isoquinolinium salt **86**, mediated by a chiral phosphite catalyst **87** (Motaleb et al., 2018).

In 2018, Maity et al. presented an example of kinetic resolution in the aerobic visible-light induced oxidation of isoquinolinium salt **86** (Scheme 23) (Motaleb et al., 2018). The process was mediated by the TADDOL-based phosphite catalyst **87**, which led to the oxidation product (*S*)-**88** in 70% ee at 45% conversion (*s* = 12.3). The proposed reaction mechanism implies the initial formation of an adduct between **86** and organocatalyst **87**, which further converts into the organocatalyst-bound α-aminoalkyl radical intermediate. The latter furnishes the oxidation product **88** upon reaction with oxygen or superoxide anion.

## Conclusion and Outlook

The progress in enantioselective aerobic oxidations in the last decades clearly demonstrates that molecular oxygen can be considered as a viable oxidant in the asymmetric transformations, being especially attractive for the development of sustainable oxidation protocols. Importantly, an efficient way of chirogenesis must be enabled along with a complementary O_2_-activation cycle to ensure high yields, extended substrate scope, and excellent optical purity of the oxidation products. In turn, the activation of O_2_ can be achieved with either transition metal catalysis as a mainstream approach or with the help of photocatalytic methods as another direction. The use of electrosynthesis can also be foreseen (Kawamata et al., 2017; Sambiagio et al., 2017), although it does not have any asymmetric applications yet. Dual catalytic processes have been commonly utilized, as with the aid of custom-designed bifunctional ligands and organocatalysts and with an additional catalytic cycle. Despite of the promising potential of tandem catalytic processes for further application, the design of task-specific catalysts and merging two complementary catalytic cycles into a tandem process are still arduous tasks.

For the forthcoming successful developments in the field, several existing challenges must be properly addressed. Although aerobic oxidations themselves are considered as “green-by-design” processes, their safety and energy efficiency characteristics are often not satisfactory for industrial applications. The use of air and especially pure oxygen in the large-scale manufacturing is hampered by incompatibility with flammable solvents, along with accompanying problems such as heat- and mass transfers in a liquid-gas reaction media (Osterberg et al., 2015; Sterckx et al., 2019). Furthermore, non-catalytic radical autooxidation events may represent an additional obstacle (Sterckx et al., 2019). Fortunately, it is possible to attenuate the impact of these negative phenomena with the aid of modern flow chemistry techiques (Gemoets et al., 2016; Hone and Kappe, 2018). Complications for the industrial use of light-driven reactions arise from their significant running cost and high energy demands (Protti et al., 2009) that eventually results in enhanced CO_2_ emissions, especially in the case of fossil fuel as a source of energy. This problem can be attenuated and eventually circumvented by further technology advances, such as design of more energy-efficient light sources, photoreactors, and use of clean energy. As for the latter, direct use of sunlight is especially appealing (Haggiage et al., 2009; Cambié et al., 2017; Cambié et al., 2019). Although the concept is more than a century old (Ciamician, 1912), its renaissance has begun only recently.

Besides the technology-related issues outlined above, chemical diversity of the available transformations must be considerably expanded. Despite rapid progress in the field of aerobic oxidations in general, the contribution of the corresponding asymmetric reactions is rather low and mostly limited to the α-hydroxylation of reactive carbonyl compounds, epoxidations of alkenes, and oxidative kinetic resolutions of secondary alcohols. As one of the solutions, an inspiration for the development of new highly chemo- and enantioselective transformations could arise from enzymatic reactions, of which many have no artificial analogs, such as efficient enantioselective hydroxylation of inactivated C–H bonds (Chang et al., 2000), including hydrocarbons (Adam et al., 2000a; Adam et al., 2000b), and *cis*-dihydroxylation of aromatic compounds (Endoma et al., 2002; García-Urdiales et al., 2005; Boyd and Bugg, 2006).

Albeit the enantioselective oxidation reactions with molecular oxygen are still in their infancy and cannot be considered as dominant among the asymmetric oxidation methods, their sustainable design and prominent potential for environmentally benign chemical industry will certainly stimulate further research activity in the field.
